# Insulin-Like Growth Factor 2 Silencing Restores Taxol Sensitivity in Drug Resistant Ovarian Cancer

**DOI:** 10.1371/journal.pone.0100165

**Published:** 2014-06-16

**Authors:** Jurriaan Brouwer-Visser, Jiyeon Lee, KellyAnne McCullagh, Maria J. Cossio, Yanhua Wang, Gloria S. Huang

**Affiliations:** 1 Department of Obstetrics and Gynecology & Women’s Health (Division of Gynecologic Oncology), Albert Einstein College of Medicine and Montefiore Medical Center, Bronx, New York, United States of America; 2 Department of Pathology, Albert Einstein College of Medicine and Montefiore Medical Center, Bronx, New York, United States of America; 3 Department of Molecular Pharmacology, Albert Einstein College of Medicine, Bronx, New York, United States of America; 4 Albert Einstein Cancer Center, Albert Einstein College of Medicine, Bronx, New York, United States of America; Baylor College of Medicine, United States of America

## Abstract

Drug resistance is an obstacle to the effective treatment of ovarian cancer. We and others have shown that the insulin-like growth factor (IGF) signaling pathway is a novel potential target to overcome drug resistance. The purpose of this study was to validate IGF2 as a potential therapeutic target in drug resistant ovarian cancer and to determine the efficacy of targeting IGF2 *in vivo*. An analysis of The Cancer Genome Atlas (TCGA) data in the serous ovarian cancer cohort showed that high IGF2 mRNA expression is significantly associated with shortened interval to disease progression and death, clinical indicators of drug resistance. In a genetically diverse panel of ovarian cancer cell lines, the IGF2 mRNA levels measured in cell lines resistant to various microtubule-stabilizing agents including Taxol were found to be significantly elevated compared to the drug sensitive cell lines. The effect of IGF2 knockdown on Taxol resistance was investigated *in vitro* and *in vivo*. Transient IGF2 knockdown significantly sensitized drug resistant cells to Taxol treatment. A Taxol-resistant ovarian cancer xenograft model, developed from HEY-T30 cells, exhibited extreme drug resistance, wherein the maximal tolerated dose of Taxol did not delay tumor growth in mice. Blocking the IGF1R (a transmembrane receptor that transmits signals from IGF1 and IGF2) using a monoclonal antibody did not alter the response to Taxol. However, stable IGF2 knockdown using short-hairpin RNA in HEY-T30 effectively restored Taxol sensitivity. These findings validate IGF2 as a potential therapeutic target in drug resistant ovarian cancer and show that directly targeting IGF2 may be a preferable strategy compared with targeting IGF1R alone.

## Introduction

Ovarian cancer is the leading cause of gynecologic cancer death in the United States. Since the mid-1990s, the standard treatment for ovarian cancer is surgical cytoreduction and systemic chemotherapy, usually Taxol and platinum [1]. However, the majority of patients eventually succumb to recurrent, progressive disease due to resistance to chemotherapy.

In addition to its well-established roles in development and growth, aging, and carcinogenesis [2]–[6], the insulin-like growth factor (IGF) signaling pathway has recently been implicated in drug resistance [7]–[11]. As we previously reported, upregulation of insulin-like growth factor 2 (IGF2) is an acute cellular response to Taxol treatment, and its expression modulates the response to Taxol in drug resistant ovarian cancer cell lines. [7]. Since Taxol is used in the first-line treatment of ovarian cancer, we hypothesized that high IGF2 would be associated with intrinsic clinical drug resistance, manifesting as decreased time to disease progression/recurrence in patients. Supporting this hypothesis, our prior study using a tissue microarray approach indicated that high IGF2 expression in ovarian tumor tissue was indeed predictive of shortened interval to progression/recurrence.

Based on these laboratory and clinical data, we determined that IGF2 is a potential therapeutic target to ameliorate drug resistance in ovarian cancer, but to our knowledge, no prior *in vivo* validation studies have been done. Therefore, as described herein, we performed studies to evaluate if Taxol resistance could be overcome *in vivo* by targeting IGF2. We examined the impact of IGF2 knockdown on not only Taxol’s effects, but also the response to non-taxane microtubule interacting drugs, and other drugs commonly used in the treatment of ovarian cancer. To confirm the clinical relevance of our findings, an analysis of the serous ovarian cancer cohort of The Cancer Genome Atlas (TCGA) was done to evaluate the relation of IGF2 expression and clinical outcomes.

## Materials and Methods

### Ethics Statement

All animal experiments were done with the approval of the Institutional Animal Care and Use Committee (Protocol 20130604) of the Albert Einstein College of Medicine of Yeshiva University. The Institutional Animal Welfare Assurance (A3312-01) for this facility is fully accredited by the Association for the Assessment and Accreditation of Laboratory Animal Care (AAALAC) since February 22, 1983. Animals were cared for as per the Animal Welfare Act and the NIH “Guide for the Care and Use of Laboratory Animals”.

### Cell Lines and Reagents

The ovarian carcinoma cell lines A2780 [12] and HEY [13] (kind gifts from Dr. Susan Band Horwitz), and the ovarian carcinoma cell line NIH:OVCAR8 [14] (a kind gift from Dr. David Goldman) were grown in RPMI-1640 (Life Technologies) with 10% Fetal Bovine Serum (Life Technologies) and 1% Penicillin/Streptomycin (Life Technologies) at 37C with 5% CO_2_. All drug resistant cell lines were generated by the authors, except HEY-Epo8 (a gift from Dr. Susan Band Horwitz) that was developed by Dr. C-P Huang Yang using epothilone B [15]. The Taxol-resistant HEY-T30 cell line was generated from HEY cells, as described previously [7]. The A2780-T15 cell line was generated similarly from A2780 using Taxol selection but in the continuous presence of 15 µM verapamil (Sigma). A2780-B20 and HEY-B20 were selected for resistance to ixabepilone (Ixempra Bristol-Myers Squibb), and OVCAR8-D30 to discodermolide. Cell lines were authenticated using the Genemarker 10 kit (Promega). Resistant cell lines were matched to their sensitive lines and to published data when available. Cell lines were routinely screened for mycoplasma with MycoAlert (Lonza). Cells were cultured in drug-free media for at least 18 hours prior to experiments except A2780-T15, which was grown in the presence of 0.5 nM Taxol. Clinically formulated Taxol (Hospira) was diluted 6-fold in 5% dextrose water (Hospira) to a final concentration of 1 mg/ml for xenograft experiments. NVP-AEW541, a small molecular weight kinase inhibitor of IGF1R, was provided by Novartis Pharma AG [16]. A monoclonal antibody to IGF1R, IMC-A12 (Cixutumumab) was provided by Imclone, a fully owned subsidiary of Eli Lilly and Company.

### IGF2 Gene Expression Analysis in Clinical Samples of Ovarian Cancer

The cBioPortal for Cancer Genomics was used to access the gene expression and clinical data from the Cancer Genome Atlas Project (TGCA) [17]. The query was performed using All Complete Tumors of the Ovarian Serous Cystadenocarcinoma (TCGA, Nature 2011) dataset, which includes 489 cases of high-grade serous ovarian cancer. For IGF2 mRNA Expression Z-scores, a threshold of 1.6 standard deviations above the mean defined the IGF-high group; all other cases were included in the IGF2-normal group.

### Quantitative PCR

Cell lysates were homogenized using Qiashredder columns (Qiagen Inc., Valencia, CA) and total RNA was isolated by RNeasy Mini Kit (Qiagen). RNA concentration and purity were evaluated using a NanoDrop spectrophotometer (Fisher Thermo Scientific), showing OD 260/280 ratio range of 2.03–2.11. RNA integrity was sampled using an Agilent Bioanalyzer (Agilent Technologies), showing a RIN score range of 9.8 to 10. Complementary DNA was made by performing reverse transcription (RT) using the SuperScript VILO cDNA Synthesis Kit (Life Technologies) according to manufacturer’s instructions, using 1 µg total RNA for all cell lines except A2780, A2780-T15 and A2780-B20, for which 2 µg total RNA was used. Quantitative real-time PCR was performed using an Eppendorf Mastercycler ep *realplex2* using a 3-step method (95C 10 min; followed by 40 cycles of 95C for 10 sec, 60C for 20 sec, 72C for 20 sec; then for melting curve 95C 15 sec, 60C to 95C over 20 min). Each reaction utilized 1/20th of the cDNA reaction, forward and reverse primers at a final concentration of 200 nM, and PowerSYBR (Applied Biosystems, Foster City, CA) diluted in Ultrapure water (Life Technologies) to 1X final concentration in a final reaction volume of 10 µL. Exon junction-spanning primers (Table S1) were validated by analysis of melting curves and amplification efficiency. An mRNA expression score was calculated using the formula 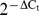 * 1000, where ΔC_t_ is the difference in C_t_ (cycle threshold) between the gene of interest and the internal normalization gene, *PPIB* (peptidylprolyl isomerase B; cyclophilin B). *PPIB* was verified to have stable mRNA expression levels in the parental, drug resistant, and transfected ovarian cell lines including: HEY, HEY-T30, HEY-B20, HEY-T30 shIGF2-p, HEY-T30 shIGF2-v, HEY-T30 shScrambled, A2780, A2780-T15, A2780-B20. The mean difference in C_t_ values when evaluating *PPIB* expression in pairwise comparisons of these cell lines was 0.2 (95% confidence interval of −0.1 to 0.5). Fold-change in relative mRNA expression was calculated using the 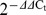 method [7]. Genomic DNA was isolated with the AllPrep mini kit (Qiagen). Primers were designed to span an intron-exon junction and were validated by melting and efficiency curve analysis. Albumin (*ALB*) was used for internal normalization. By array comparative genomic hybridization, HEY cells were found to have two copies of the *IGF2*, *ABCB1* and *ALB* genes (data not shown). Copy number variation was determined from qPCR of genomic DNA using the 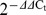 method and setting HEY to 2 copies [18].

### Proliferation Kinetics and Cytotoxicity Assays

For proliferation kinetics, cells were seeded in 6-well plates. Every 24 hours, duplicate wells were counted using a Scepter Counter (Millipore). For cytotoxicity assays, cells were seeded in a 96 well plate. After 24 hours, cells were treated with serial dilutions of the indicated drug, with a fixed concentration of 1 µM NVP-AEW541 or 10 µg/ml IMC-A12 if indicated. After 72 hours of treatment, the relative cell number in each well was determined using the Sulforhodamine B assay [7]. The IC_50_ is the drug concentration corresponding to a 50% decrease in cell number calculated using the dose-effect curve.

### ELISA for Receptor Phosphorylation

Cells were grown in complete media for 24 hours, then serum-starved for 12 hours before stimulation for 10 minutes with recombinant IGF2 (50 ng/mL; Abcam) or insulin (50 nM; Sigma), with or without pretreatment of cells with NVP-AEW541 (1 µM) or IMC-A12 (10 µg/ml) for 2 hours. Cells were lysed using NP40-based lysis buffer with PhosSTOP (Roche) and protease inhibitor cocktail (Sigma). After protein quantitation, the DuoSet IC Human Phospho-IGF-1 R and Phospho-Insulin R (R&D systems) were used according to the manufacturer’s instructions.

### Western Blot Analysis of Protein Expression

For analysis of phosphorylated and total AKT and ERK, equal amounts of protein (15 to 20 µg, depending on the experiment) were loaded in each lane on a tris-glycine gel and transferred to nitrocellulose membranes, which were blocked and incubated overnight with the indicated primary antibodies diluted 1∶1000 in blocking buffer (LI-COR Biosciences), except GAPDH used at 1∶5000 dilution. Primary antibodies used were: PhosphoAKT (Thr308) (Cell Signaling Technology 4056), PhosphoAKT (Ser473) (Cell Signaling Technology 4060), AKT1 (Cell Signaling Technology 2938), PhosphoERK1/2 (Thr202/Tyr204) (Cell Signaling Technology 4370), ERK 1/2 (Cell Signaling Technology 4695), GAPDH (Cell Signaling Technology 2118), IGF2 (Abcam AB9574). After secondary antibody (1∶10 000 anti-rabbit, LI-COR Biosciences 926–32211) incubation, membranes were scanned on an Odyssey infrared imaging system (LI-COR Biosciences) at 800 nm wavelength. Densitometry was done on the TIFF files measuring integrated density using ImageJ, correcting for background and normalizing to Ponceau staining of the respective lane.

### Therapeutic Studies in Xenograft-bearing Mice

Female athymic nude mice (Harlan) between 6 and 8 weeks old were injected subcutaneously with one million of the indicated cells, suspended in 100 µl OptiMem. Tumor size was measured using digital calipers and volume calculated using the formula: (length * width^2^)/2. Mice were treated as described in the figure legends. Mice were euthanized by isoflurane anesthesia followed by cervical dislocation, when their xenograft reached 20 mm diameter, or at the specified time points for xenograft analysis. Hematoxylin and eosin stained xenograft sections were evaluated by the study pathologist (Y.W.). Immunohistochemistry for IGF2 (Abcam ab9574) was done on formalin-fixed paraffin-embedded sections as described previously for tumor tissues [7] and scored by the study pathologist (Y.W.).

### IGF2 and IR Knockdown

The IGF2 and IR-targeting siRNA oligonucleotides were purchased from Life Technologies. Using RNAiMax Lipofectamine (Life Technologies), reverse transfection was performed as previously described [7] in parallel with non-targeting siRNA transfection and mock transfection controls. RNA was harvested at 48 hours post transfection to confirm knockdown by RT-qPCR (Fig. S2A/D in File S1). For HEY-T30 cells, results from the first IGF2 siRNA oligonucleotide sequence tested was shown in a prior publication [7]; validation of these findings was done for this manuscript using two new unique oligonucleotide sequences arbitrarily named siIGF2-1 and siIGF2-2. For A2780-T15 cells, two oligonucleotide sequences were used, corresponding to the first siRNA used in HEY-T30 (arbitrarily named siIGF2-3) as well as the new oligonucleotide siIGF2-1. For stable knockdown using the BLOCK-iT Inducible H1 Lentiviral RNAi System (Life Technologies), vectors were designed to express an shRNA targeting IGF2, or a non-targeting control (shScrambled). HEY-T30 cells were transfected with the plasmid directly or with lentiviral particles, then selected with Zeocin (Life Technologies). Clones of plasmid-transfected cells and lentiviral-transfected cells were screened for IGF2 knockdown. The clone with the lowest IGF2 mRNA expression from each group was used for subsequent experiments.

### Cell Cycle and Apoptosis Analysis

Cells were treated as described in the figure legends. After 16 hours, adherent and floating cells were collected. For cell cycle analysis, cells were fixed with 70% cold ethanol for 1 hour, then incubated with 20 µg/ml propidium iodide (Sigma) and 100 µg/ml RNase (Fisher Thermo Scientific) in PBS. For apoptosis analysis, the Violet Ratiometric Membrane Asymmetry Probe/Dead Cell Apoptosis Kit (Life Technologies) was used according to the manufacturer’s protocol. A violet excitable dye 4′-N,N-diethylamino-6-(N,N,N- dodecyl-methylamino-sulfopropyl)-methyl-3-hydroxyflavone (F2N12S) was used for the detection of membrane asymmetry changes that occur during early apoptosis, resulting in a decreased ratio of 585 nm to 530 nm emission. Simultaneously, SYTOX(R)AADvanced was used as a dead cell stain. Using unstained cells and control untreated cells, gating of quadrants was determined; live, apoptotic, and dead cells were quantified using FlowJo (Treestar).

### Drug Efflux Analysis

Two hundred thousand HEY and HEY-T30 cells were resuspended in 1 ml warm PBS +5% FBS. All incubation steps were done at 37C in the cell culture incubator. Verapamil (15 µM) or NVP-AEW541 (1 µM) was added and the samples incubated for 1 hour. Then 50 µM of Tubulin Tracker Green reagent (Oregon Green 488 Taxol, bis-acetate) (Life Technologies) was added and the samples incubated for 45 minutes. Cells were pelleted then resuspended in PBS +5% FBS, with verapamil or NVP-AEW541 if indicated, and incubated for an additional 20 minutes, washed in PBS and stained with TO-PRO-3 (Life Technologies). Cells were immediately analyzed using a FACSCanto II (BD) and FlowJo; TO-PRO-3 positive cells (dead cells) were gated out and the remaining cells were graphed to determine labeled Taxol retention [19].

### Statistical Analysis

For each analysis, the data was from at least two independent experiments. The numbers of replicates per experiment are described in the figure legends. Differences between means of two groups were analyzed using the t-test. For differences between three or more groups, one-way ANOVA with a Bonferroni post-test was used. For analysis of two independent variables, two-way ANOVA with a Bonferroni post-test was used. For survival analysis, the logrank test was used. All P values are two-tailed and P<0.05 was considered statistically significant.

### Supplemental Methods

File S2 contains the materials and methods used to generate the β-tubulin mutation data (shown in Fig. S1 of File S1) and the IGF2 Western blot data (shown in Fig. S2E of File S1).

## Results

**Figure 1:**
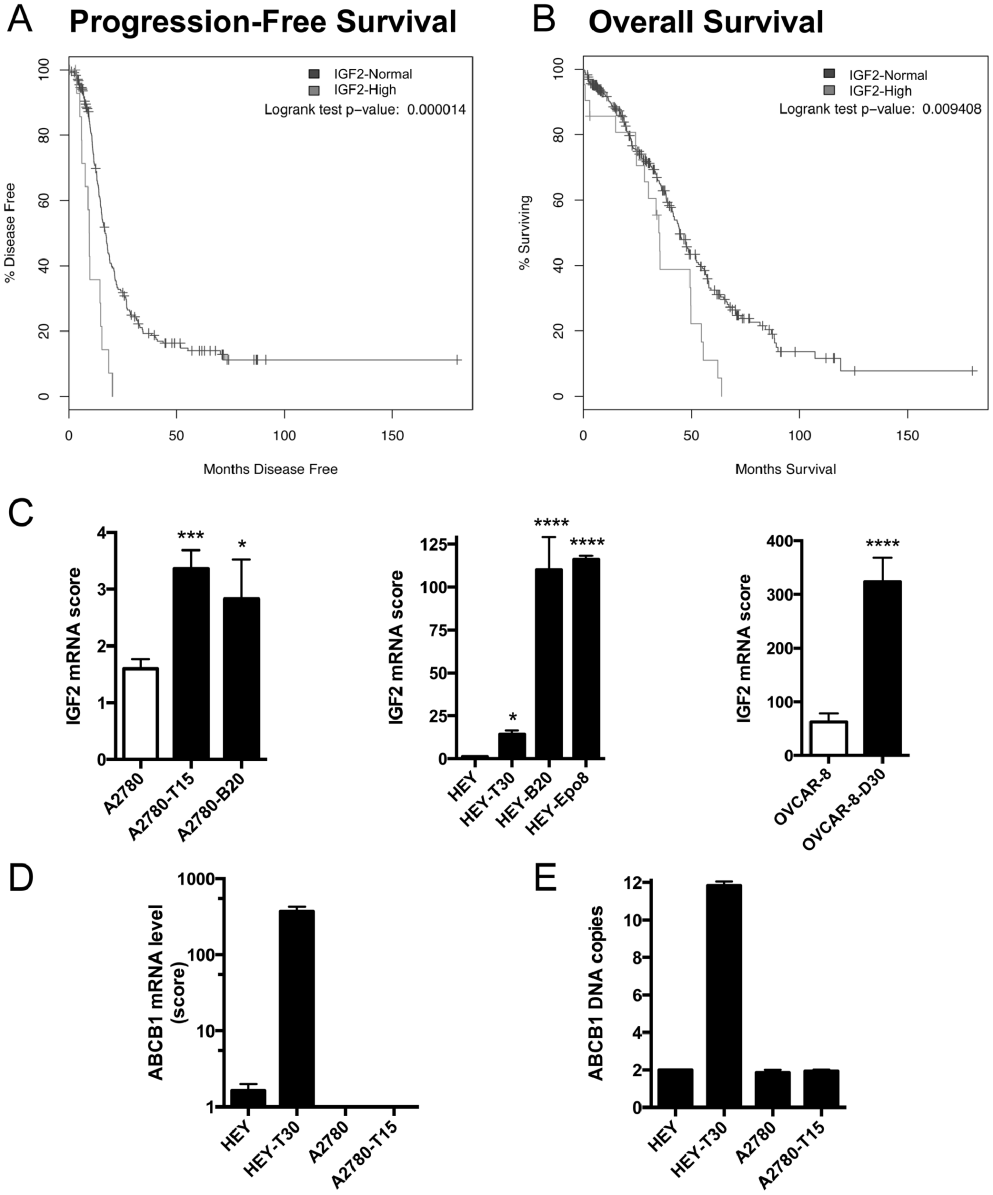
High IGF2 expression is associated with early recurrence, poor survival, and drug resistance. IGF2 expression and ovarian cancer survival. Using the cBioPortal for Cancer Genomics to analyze the data from the Cancer Genome Atlas study of ovarian serous cystadenocarcinoma, we compared the progression-free survival and overall survival in patients with high tumor levels of IGF2 mRNA (greater than 1.6 standard deviations above the mean; gray line) and those with normal tumor levels of IGF2 mRNA (all other patients; black line). Patients with high IGF2 mRNA levels had significantly shortened progression-free survival (A) and overall survival (B). (C) IGF2 expression in sensitive and resistant cell lines. By RT-qPCR, we measured the IGF2 mRNA level in six drug resistant ovarian carcinoma cell lines and their three cell lines of origin. All drug resistant cell lines have significantly higher IGF2 mRNA expression compared to their sensitive cell line of origin. Bars show the mean±SEM of IGF2 mRNA expression score for at least two independent experiments for each cell line, each done in triplicate, and the symbol above the bar indicates the statistical significance comparing that resistant cell line with its parental counterpart; *p<0.05, ***p<0.001, ****p<0.0001 by One-way ANOVA with Bonferroni posttest. (D) ABCB1 expression. ABCB1 mRNA expression levels were measured by RT-qPCR in HEY, HEY-T30, A2780 and A2780-T15. HEY-T30 has higher ABCB1 mRNA expression compared to the other cell lines. Bars show the mean±SEM of ABCB1 mRNA expression score for at least four independent experiments for each cell line, each done in triplicate, (E) ABCB1 DNA copy number. By qPCR performed on genomic DNA, HEY-T30 has a six-fold increase in ABCB1 DNA copy number indicating gene amplification. Bars show the mean±SEM of two independent experiments, each done in triplicate.

Our prior study of IGF2 protein expression using a tissue microarray of epithelial ovarian tumors indicated that high tissue expression levels of IGF2 were associated with a shortened interval to progression/recurrence [7]. Since then, gene expression data from 489 clinically-annotated stage II-IV high-grade serous ovarian cancer samples of The Cancer Genome Atlas project (TCGA) were made available through the cBioPortal for Cancer Genomics [17]. Using this data portal, we tested a relationship of IGF2 expression and intrinsic drug resistance, wherein high IGF2 was defined as 1.6 SD above the mean, corresponding to 94–95th percentile in a normal distribution. By using this cut score, the size of the IGF2-high group approximates the fraction of ovarian cancer patients expected to have intrinsically drug resistant tumors, manifested by progression during or shortly after completing chemotherapy. In our analysis of the TCGA data, we indeed found that high IGF2 mRNA expression in tumor tissue significantly correlated with a shortened interval to progression/recurrence, i.e. progression-free survival (Fig. 1A). Median progression-free survival in this group was <12 months, indicative of intrinsic drug resistance. High IGF2 mRNA expression also correlated with significantly shortened overall survival (Fig. 1B). Multivariate analysis was not enabled by this data portal, however in our prior publication we noted an association of IGF2 expression with higher stage and grade [7].

We previously showed that IGF2 mRNA levels increase during acute Taxol treatment, and we hypothesized that upregulation of IGF2 is associated with persistent surviving cells giving rise to drug resistant recurrent disease. To study drug resistance in the laboratory, Taxol, as well as non-taxane microtubule-stabilizing agents (MSAs), ixabepilone, discodermolide, and epothilone B, were used to develop six distinct resistant cell lines. As determined by real-time PCR, all MSA-resistant cell line models had elevated IGF2 mRNA expression relative to their sensitive parental line (Fig. 1C). Since Taxol is by far the most clinically used among these drugs, subsequent experiments focused on the Taxol resistant cell lines HEY-T30 and A2780-T15.

We evaluated the drug resistant cell lines for other mechanisms of Taxol resistance [20], [21]. HEY-T30 has ABCB1 mRNA over expression (Fig. 1D) associated with genomic amplification of *ABCB1* (Fig. 1E), while HEY has no copy number change of *ABCB1*. These findings were confirmed by array comparative genomic hybridization (data not shown). A2780-T15 was selected in the presence of verapamil and does not overexpress ABCB1 (Fig. 1D), and neither A2780-T15 nor A2780 has DNA copy number change of *ABCB1* (Fig. 1E). We found no significant correlation between IGF2 and ABCB1 expression in our panel of cell lines (Fig. S3 of File S1). DNA sequencing revealed that A2780-T15 has a mutation (Glycine at position 360 is changed to an Aspartic acid) in the Taxol-binding pocket of β-tubulin (Fig. S1A and S1B of File S1), while HEY-T30 does not have a mutation in β-tubulin.

**Figure 2:**
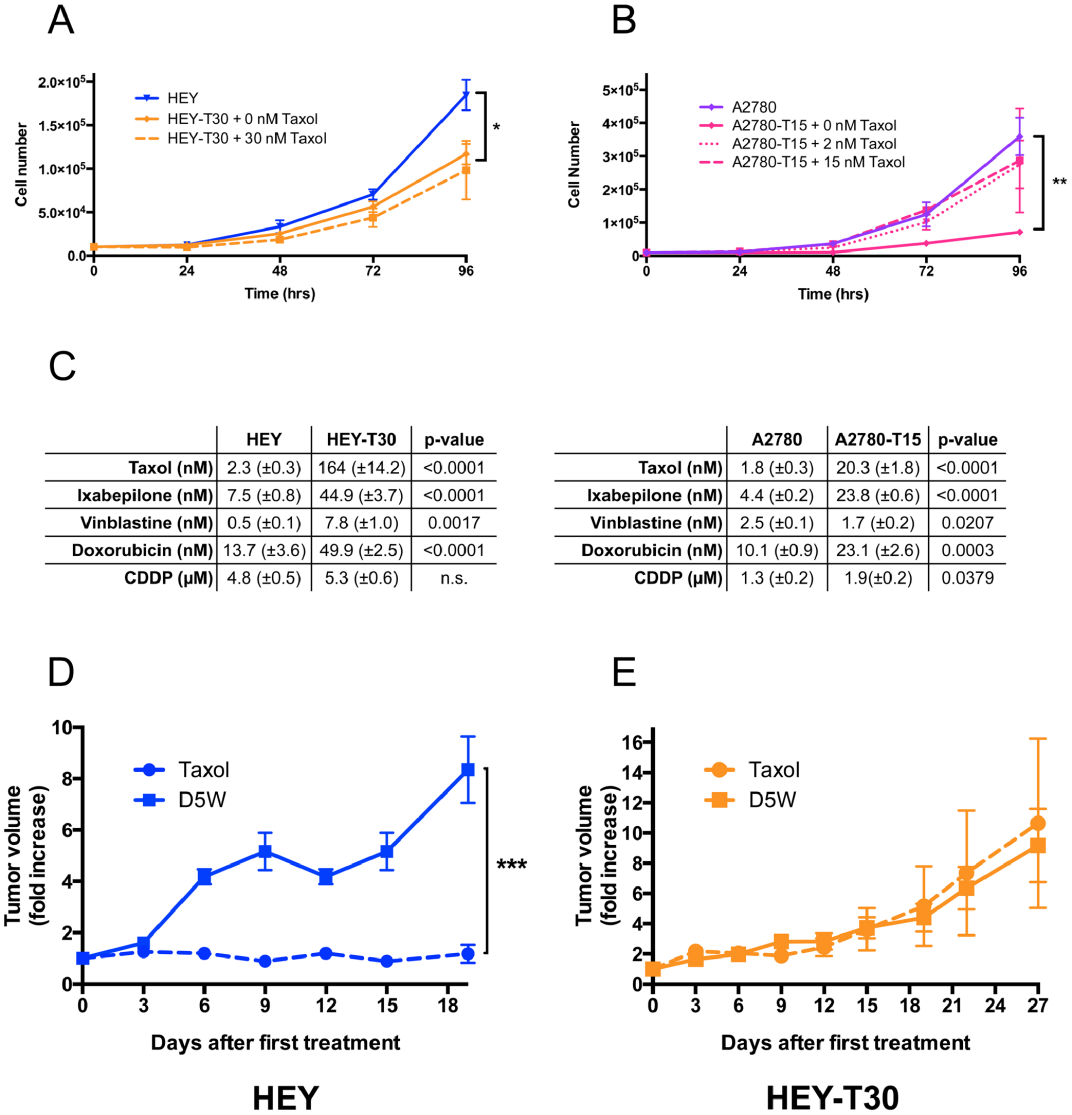
Characterization of the drug resistance phenotype. Proliferation kinetics of cell lines. Cells were grown in complete media with the indicated concentration of Taxol in 6-well dishes, and cells from duplicate wells counted every 24 hours. (A) HEY-T30 cells in the presence or absence of 30 nM Taxol proliferate more slowly than HEY cells (p<0.05, Repeated Measures Two-way ANOVA, Bonferroni posttest). (B) A2780-T15 in the presence of 2 nM and 15 nM Taxol grow similarly to A2780. However, in the absence of Taxol, A2780-T15 proliferates more slowly, suggesting Taxol dependence (p<0.01, Two-way ANOVA, Bonferroni posttest). Each growth curve represents the mean of at least two independent experiments each done in duplicates, each point is represented as the mean±SEM. (C) IC_50_’s of chemotherapeutic drugs in sensitive and resistant cell lines. In 96-well plates, cells were treated with serial dilutions of the indicated drugs and the concentration of 50% proliferation inhibition (IC_50_) determined using the SRB cytotoxicity assay. HEY-T30 are cross-resistant to ixabepilone and to vinblastine. There is modest cross-resistance to doxorubicin but not to CDDP. A2780-T15 are cross-resistant to ixabepilone, and modestly cross-resistant to doxorubicin and CDDP, but more sensitive to vinblastine. Data are presented as the mean±SEM of at least three independent experiments performed with six replicates. P-values calculated by unpaired t-test. (D, E) Comparison of HEY-T30 and HEY xenograft response to Taxol. Following subcutaneous injection of HEY-T30 or HEY cells, mice were divided into treatment groups of 6 animals each. When the average xenograft volume reached 120 mm^3^, Taxol treatment was administered at the maximal tolerated dose (MTD) of 20 mg/kg intraperitoneally every three days for five treatments (treatment days: 0, 3, 6, 9, 12), resulting in a cumulative dose of 100 mg/kg. Control animals received intraperitoneal injections of the diluent (5% dextrose in water; D5W) according to the same schedule. Tumors were measured every three days, and mean tumor volumes±SEM for each group shown at each time point. HEY xenografts responded to Taxol treatment, which potently suppressed tumor growth (Fig. 2D; dashed blue line). HEY-T30 xenografts did not respond to Taxol treatment (Fig. 2E; dashed orange line) and grew at a similar rate as vehicle-treated HEY-T30 xenografts. HEY Taxol vs HEY D5W on day 19, p<0.001; unpaired t-test.

HEY-T30 proliferated similarly in Taxol-free media or media containing low concentrations of Taxol (≤30 nM) (Fig. 2A). A2780-T15 proliferated much more slowly in Taxol-free media than in media containing Taxol (≤15 nM) (Fig. 2B), suggesting possible Taxol dependent-growth associated with its β-tubulin mutation. When low-dose Taxol was present, A2780-T15 proliferated at a similar rate as A2780. The resistant cell lines showed cross-resistance to ixabepilone, an MSA that shares a common β-tubulin binding site with Taxol. Expanded profiling of HEY-T30 (Fig. 2C, left panel) showed resistance to the microtubule-destabilizing drug vinblastine and resistance to doxorubicin, but similar sensitivity to cisplatin, compared to parental HEY. A2780-T15 (Fig. 2C, right panel) was cross-resistant to both doxorubicin and CDDP, but was more sensitive to vinblastine. Hypersensitivity to microtubule-destabilizing drugs has been observed previously in another drug resistant cell line harboring a β-tubulin mutation that was associated with dependency on microtubule-stabilizing drugs for proliferation [22].

A critical step in translating laboratory findings into potential therapies for patients is to conduct *in vivo* testing using animal models. Since A2780-T15 cells were dependent on Taxol for their growth but HEY-T30 were not, we evaluated HEY-T30 as a xenograft model for *in vivo* testing of therapeutic strategies targeting drug resistance. We administered Taxol using the previously determined maximum-tolerated dose (MTD; cumulative dose of 100 mg/kg) [23] to evaluate drug resistance *in vivo*. Parental HEY xenograft growth was effectively suppressed by Taxol, indicating sensitivity to Taxol (Fig. 2D). In contrast, Taxol failed to inhibit HEY-T30 xenograft growth compared to vehicle (5% dextrose in water; D5W) treatment, indicating drug resistance (Fig. 2E).

**Figure 3:**
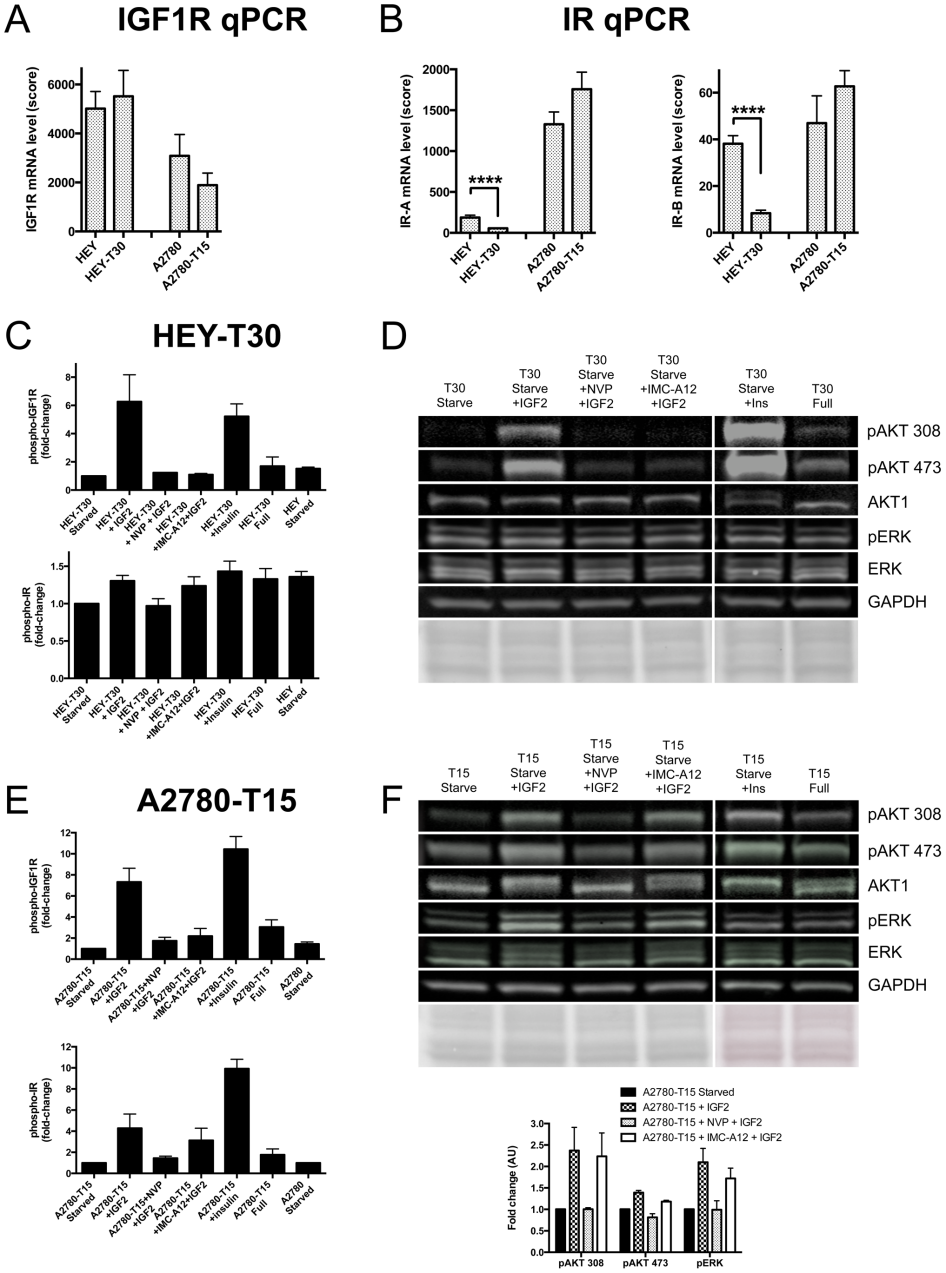
Effects of ligand stimulation and targeted therapeutics on IGF1R and IR expression and phosphorylation. (A) IGF1R mRNA of sensitive and resistant cell lines. Measured by RT-qPCR (n = 5 independent experiments each done in triplicate), IGF1R mRNA levels are similar when resistant cell lines are compared to their parental cell lines of origin. Bars show the mean±SEM. (B) IR mRNA of sensitive and resistant cell lines. Measured by RT-qPCR (n = 5 independent experiments each done in triplicate) IR-A and IR-B mRNA levels in HEY-T30 are significantly decreased when compared to HEY, while similar levels are observed when comparing A2780-T15 to A2780. Bars show the mean±SEM, ****p<0.0001; unpaired t-test. (C) Phospho-IGF1R and phospho-IR ELISA in HEY-T30. Cells were starved overnight, incubated with 1 µM NVP-AEW541 or 10 µg/ml IMC-A12 for 2 hours, stimulated with 50 ng/ml IGF2 or 50 nM insulin for 10 minutes, lysed, and levels of phosphorylated IGF1R and IR determined by ELISA. Both IGF2 and insulin caused 5- to 6-fold-change increased levels of phospho-IGF1R that could be suppressed by NVP-AEW541 or IMC-A12. The changes in phospho-IR after stimulation with IGF2 or insulin were much smaller (fold-change <1.5). Bars depict mean±SEM fold-change phosphorylation levels of at least two independent experiments, each done in duplicate. (D) Phospho-AKT and phospho-ERK Western blot. Cell lysates prepared as described in (C) were used for immunoblotting using phosphorylation-specific AKT and ERK antibodies. IGF2-induced AKT phosphorylation at threonine 308 and serine 473 was suppressed by NVP-AEW541 and IMC-A12, while total AKT was unchanged. No effect was seen on phospho-ERK levels. One representative experiment of two independent experiments is shown. Equal protein loading was confirmed by Ponceau staining and GAPDH immunoblotting. (E) Phospho-IGF1R and phospho-IR ELISA in A2780-T15. Cells were prepared similarly to (C). IGF2 and insulin caused 7-fold and 10-fold increased levels of phospho-IGF1R, respectively, and this could be suppressed by NVP-AEW541 or IMC-A12. Levels of phospho-IR after IGF2 or insulin stimulation also increased, 4-fold and 9-fold, respectively. Suppression of phospho-IR with NVP-AEW541 was more effective than with IMC-A12, at these concentrations. Bars depict mean fold-change phosphorylation levels±SEM of at least two independent experiments, each done in duplicate. (F) Phospho-AKT and phospho-ERK Western blot. IGF2-induced AKT and ERK phosphorylation was suppressed by NVP-AEW541 but not by IMC-A12. One representative experiment of two independent experiments is shown. Quantitation by densitometry is shown in the accompanying graph, where bars depict the mean expression±SEM (from two independent experiments) of each of the indicated phospho-proteins, normalized to starved unstimulated cells.

To strategize how to target IGF2-mediated drug resistance, we examined receptor expression and activation in the drug resistant and sensitive cell lines. Previously published studies indicated that IGF2-binding induces autophosphorylation of IGF1R and the insulin receptor isoform A, IR-A [24], [25], resulting in receptor activation and downstream signaling to effector molecules such as AKT. In addition, high IR-A/IGF1R expression was associated with resistance to anti-IGF1R therapies, as signaling through IR-A can circumvent dependency on IGF1R [26]. We determined that IGF1R mRNA expression was higher in HEY and HEY-T30, compared with A2780 and A2780-T15, without significant differences between the paired sensitive and resistant lines (Fig. 3A). As shown in Fig. 3B, IR-A levels were lower in HEY and HEY-T30 compared to A2780 and A2780-T15. Real time PCR data was also analyzed with correction for differences in amplification efficiency between primer sets (Table S1 of File S1), with similar findings. By Western blot, IGF1R and IR protein expression corresponded to mRNA levels (data not shown). Thus, HEY and HEY-T30 had lower IR-A/IGF1R ratios than A2780 and A2780-T15.

Receptor activation and downstream signaling induced by IGF2 was evaluated. Phosphorylation of IGF1R increased over 5-fold after IGF2 or insulin stimulation in HEY-T30 (Fig. 3C upper panel). Much smaller changes (<1.5-fold) were observed in IR phosphorylation compared to IGF1R phosphorylation levels after IGF2 or insulin stimulation of HEY-T30 (Fig. 3C lower panel). Downstream, we observed increased phosphorylation of AKT at serine 473 and threonine 308 after IGF2 or insulin stimulation, but no changes in ERK phosphorylation (Fig. 3D). Equal protein loading was confirmed by both Ponceau staining of the membrane and incubation with a GAPDH antibody. IGF2-stimulated phosphorylation of IGF1R and AKT were potently inhibited by either the small molecule IGF1R inhibitor NVP-AEW541 or the anti-IGF1R monoclonal antibody IMC-A12.

Compared to HEY and HEY-T30, A2780 and A2780-T15 had significantly higher IR-A expression (Fig. 3B). In A2780-T15, IGF2 or insulin stimulation significantly increased phosphorylation of both IGF1R and IR (Fig. 3E). NVP-AEW541 inhibited phosphorylation of IGF1R and IR. IMC-A12 inhibited IGF2-induced IGF1R phosphorylation, while having minimal effect on IGF2-induced IR phosphorylation, indicating specificity for IGF1R. Downstream, NVP-AEW541 inhibited IGF2-mediated AKT and ERK phosphorylation, while IMC-A12 did not (Fig. 3F).

**Figure 4:**
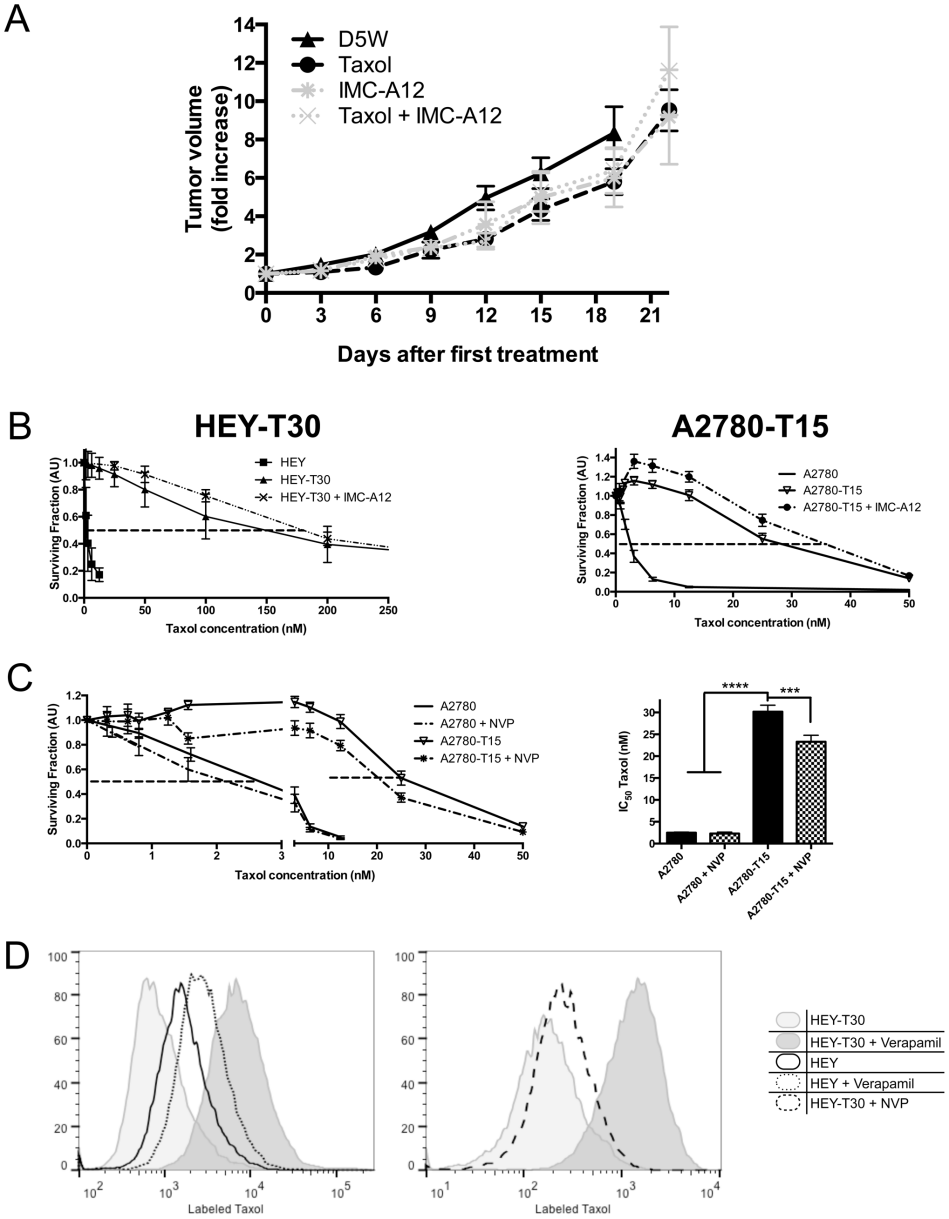
IGF1R-specific inhibition does not alter Taxol resistance. (A) Combination treatment using Taxol and IMC-A12 in mice with HEY-T30 xenografts. HEY-T30 xenografts were grown as described in Figure 2D, then treated with Taxol (black circles), according to the same dose/schedule described in Figure 2D, or with IMC-A12 (intraperitoneal injection of 40 mg/kg three times per week, grey *), or with combination treatment of Taxol and IMC-A12 (grey x). Data points show the mean tumor volume±SEM for each treatment group (n = 8 animals per group). No significant differences in tumor volume were observed with IMC-A12 or combined Taxol/IMC-A12 treatment compared to D5W or Taxol alone. This experiment was repeated a second independent time with similar results. (B) Effect of IMC-A12 on Taxol sensitivity, evaluated by SRB cytotoxicity assay. In 96-well plates, cells were treated with serial dilutions of Taxol alone (solid lines) or together with a fixed dose of 10 µg/ml IMC-A12 (dashed lines). Dose response curves show the mean of the surviving fraction±SEM of cells relative to untreated cells at the indicated Taxol concentrations (n = 7 independent experiments, each done in six replicates). No significant Taxol sensitization was observed in the presence of IMC-A12. (C) Effect of NVP-AEW541 on Taxol sensitivity, evaluated by SRB cytotoxicity assay. In 96-well plates, cells were treated with serial dilutions of Taxol alone (solid lines) or together with a fixed dose of 1 µM NVP-AEW541 (dashed lines). Dose response curves show the mean±SEM of the surviving fraction of cells relative to untreated cells at the indicated Taxol concentrations. The bar graphs depict the IC_50_ of Taxol for the indicated cell lines and drug treatments, and show the mean±SEM calculated from at least four independent experiments (each done in six replicates). NVP-AEW541 sensitizes A2780-T15 to Taxol. ***p<0.001, ****p<0.0001; One-way ANOVA with Bonferroni posttest. (D) ABCB1 (p-glycoprotein) function in HEY and HEY-T30 in the presence and absence of NVP-AEW541. Cells were incubated with labeled Taxol (Oregon Green 488 Taxol, bis-acetate) in the presence or absence of NVP-AEW541 or verapamil. The ABCB1-overexpressing HEY-T30 cells (light gray shaded graph) retain less labeled Taxol than HEY cells (unfilled black outline). Verapamil, a known p-glycoprotein inhibitor, markedly increases the retention of labeled Taxol in HEY-T30 cells (and to a lesser degree in HEY cells), as demonstrated by the rightward shift of the peaks. Shown in the right panel with the dashed black line, treatment with 1 µM NVP-AEW541 does not appear to affect retention of labeled Taxol in HEY-T30 cells. Two independent experiments were done with similar results.

Previously, we showed that IGF pathway inhibition overcame IGF2-mediated Taxol resistance in tissue culture [7]. To validate this finding *in vivo*, the effect of IGF pathway inhibition on Taxol resistance was evaluated HEY-T30 xenografts. Since NVP-AEW541 is not being clinically developed, we used IMC-A12 to specifically target IGF1R *in vivo,* using a validated regimen that inhibits IGF1R phosphorylation and reduces IGF1R protein levels in subcutaneous xenografts [27]. As shown in Fig. 4A, IMC-A12 alone or in combination with Taxol did not reduce tumor growth compared to vehicle or Taxol treatment. Concordant with the *in vivo* findings, SRB cytotoxicity assays showed no change in the sensitivity to Taxol in the presence of IMC-A12 compared to Taxol alone (Fig. 4B). In contrast, NVP-AEW541 sensitized HEY-T30 to Taxol, as previously shown (Fig. 3 in [7]), NVP-AEW541 also sensitized A2780-T15 to Taxol (Fig. 4C), although to a lesser degree compared to the effect in HEY-T30. Neither IMC-A12 alone nor NVP-AEW541 alone reduced cell survival or proliferation. We explored if the differential ability of NVP-AEW541 and IMC-A12 to sensitize cells to Taxol was due to IR. IR knockdown was accomplished by siRNA transfection, with knockdown efficiency ranging from 71–83% for IR-A and 73–76% for IR-B (Fig. S2A of File S1), with no significant effect on IGF1R (Fig. S2B of File S1). As shown in Figure S2C of File S1, IGF1R inhibition using IMC-A12 did not appear to affect Taxol sensitivity in HEY-T30 even after IR knockdown (hatched bars).

Since concurrent targeting of IR and IGF1R did not recapitulate the Taxol-sensitizing effect observed with NVP-AEW541, we also tested whether NVP-AEW541 altered drug efflux. As a result of p-glycoprotein-mediated drug efflux, HEY-T30 retain less labeled Taxol compared to HEY, while treatment with the p-glycoprotein inhibitor verapamil increases drug retention (Fig. 4D; left panel). In contrast, treatment with NVP-AEW541 at the 1 µM concentration used for cytotoxicity assays and signaling studies did not affect retention of labeled Taxol (Fig. 4D; right panel). Therefore, NVP-AEW541 at the concentrations used for cytotoxicity and signaling assays did not modulate drug efflux.

**Figure 5:**
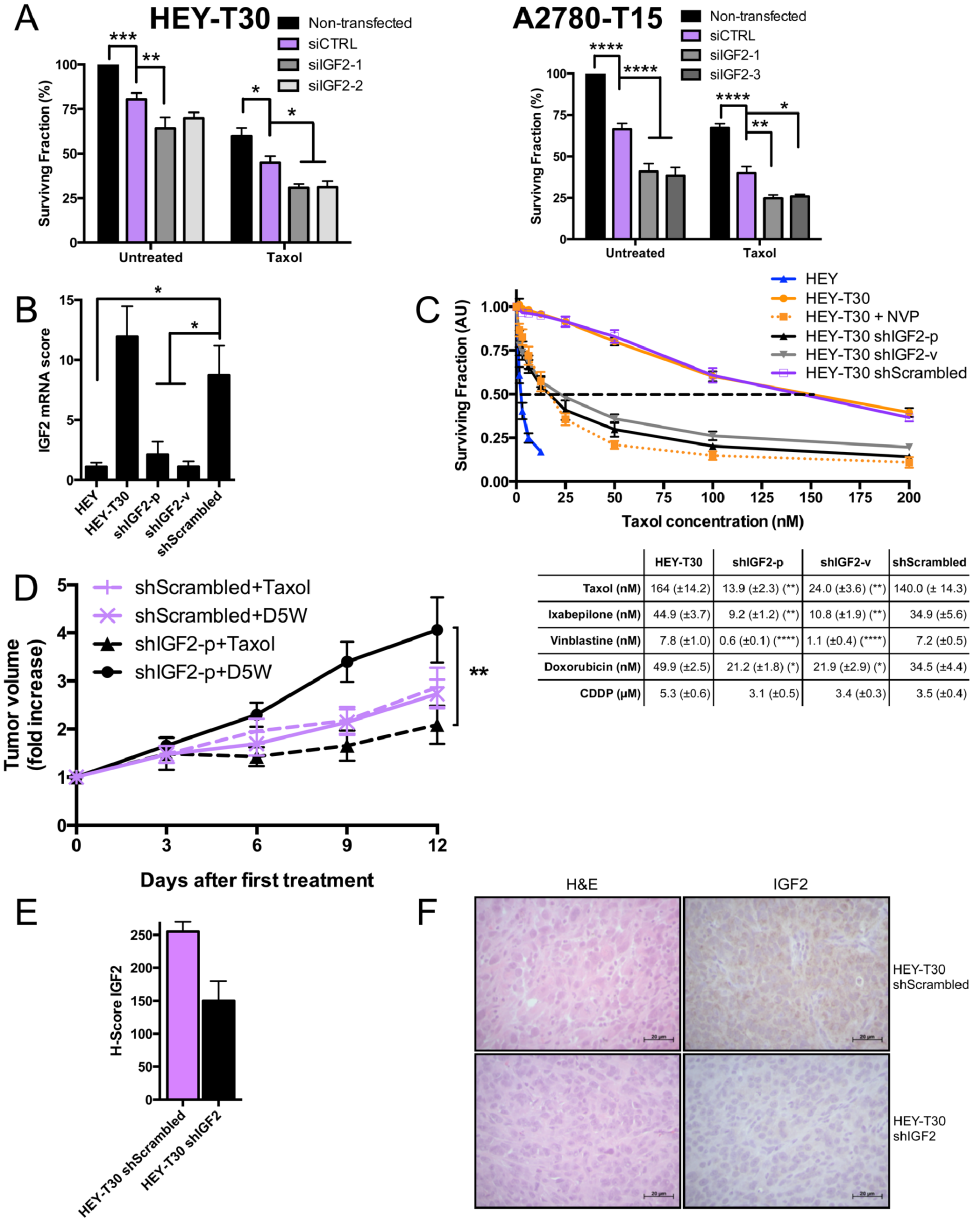
IGF2 knockdown restores Taxol sensitivity. (A) Taxol sensitivity after IGF2 knockdown by siRNA. HEY-T30 and A2780-T15 cells were transfected with control nontargeting siRNA (siCTRL) or siRNAs targeting IGF2 (siIGF2-1, siIGF2-2, siIGF2-3), and treated with DMSO or Taxol at the approximate IC_50_ (100 nM for HEY-T30 and 15 nM for A2780-T15). The surviving fraction was reduced after IGF2 siRNA transfection compared to control siRNA transfection. The effect of Taxol was significantly enhanced in both HEY-T30 and A2780-T15 by IGF2 siRNA transfection compared with control siRNA transfection. Bars represent the mean of four independent experiments (each done in duplicate)±SEM; *p<0.05, **p<0.01, ***p<0.001, ****p<0.0001; Two-way ANOVA with Bonferroni posttest. (B) IGF2 mRNA after stable knockdown with shRNA. HEY-T30 cells were transfected with shRNA targeting IGF2 by plasmid transfection (shIGF2-p) or lentiviral infection (shIGF2-v), or with a control vector containing the scrambled version of the IGF2 targeting sequence (shScrambled). Clonal stably-transfected cell lines were used for all experiments. IGF2 mRNA levels are depicted in bars±SEM. The shIGF2-p and shIGF2-v cell lines have low IGF2 mRNA levels similar to HEY, whereas HEY-T30 and shScrambled cell lines had several-fold higher mRNA expression levels. (n>6 independent experiments, each done in triplicate) *p<0.05; One-way ANOVA with Bonferroni posttest. (C) Cytotoxicity assays in cell lines with IGF2 shRNA. The indicated cell lines were treated with serial dilutions of Taxol, and sensitivity to Taxol determined by the sulforhodamine B assay. The dose-response curves are shown on the left, with the mean±SEM go the surviving fraction of cells relative to untreated cells shown at the indicated Taxol concentrations. The mean IC_50_ values±SEM for the indicated cell lines and drugs are shown in the table below. HEY T30 shIGF2-p and HEY-T30 shIGF2-v were significantly more sensitive to Taxol, ixabepilone, and vinblastine compared to control transfected HEY-T30 (shScrambled) or untransfected HEY-T30. The efficacy of IGF2 knockdown at restoring sensitivity to Taxol was similar to NVP-AEW541 treatment. Only 1.6 fold sensitization to doxorubicin and no effect on cisplatin sensitivity were observed in shIGF2 lines compared to shScrambled or untransfected HEY-T30. Asterisks denote the statistical significance when comparing the IC_50_ for the indicated shIGF2 cell line versus shScrambled, where *p<0.05, **p<0.01, ****p<0.0001; Two-way ANOVA with Bonferroni posttest. (n = 5 independent experiments each done in six replicates). (D) Xenograft growth with Taxol treatment of HEY-T30 shIGF2-p and HEY-T30 shScrambled. Female athymic nude mice were subcutaneously injected with 1 million HEY-T30 shScrambled or HEY-T30 shIGF2-p cells and tumors were allowed to grow to an average volume of 120 mm^3^. Mice were then treated with either D5W (vehicle) or Taxol at the MTD (described in 2D). Tumors were measured every three days, and data points show the mean tumor volume±SEM for each group at each time point. Two independent experiments were done for a total of 8–10 animals per group. shIGF2-p xenografts responded to Taxol treatment (triangle, dashed black line) and showed significantly tumor growth suppression compared to treatment with D5W (circle, solid black line); at day 12, **p<0.01 for shIGF2-p Taxol vs shIGF2-p D5W; One-way ANOVA with Bonferroni posttest. In contrast, HEY-T30 shScrambled xenografts did not respond to Taxol (+, dashed purple line) and continued growing at a similar rate as the vehicle-treated HEY-T30 shScrambled group (x, solid purple line). No significant difference in tumor size was observed between D5W-treated shIGF2-p and shScrambled xenografts at day 12. Representative tumor xenografts were excised on day 13 after treatment for further analysis. (E, F) IGF2 immunohistochemical staining of xenografts. Excised tumor xenografts (n = 2 animals per group) on day 13 after treatment initiation were formalin-fixed, paraffin embedded and sections stained with hematoxylin and eosin or with an anti-human IGF2 antibody. Staining was evaluated by the pathologist blinded to the groups and the mean of H-scores±SEM calculated and graphed (E). (F) Shows representative HEY-T30 shScrambled and HEY-T30 shIGF2 xenograft sections stained with hematoxylin and eosin (H&E) or with anti-IGF2 antibody (right panels). HEY-T30 shIGF2-p xenografts showed lower IGF2 expression when compared to HEY-T30 shScrambled.

Our prior data suggested that directly targeting IGF2 would be an efficacious approach to restoring Taxol sensitivity [7]. First using several siRNA sequences to IGF2 (siIGF2), we evaluated the effect of IGF2 knockdown in HEY-T30 and A2780-T15. Transfection with each of the unique siIGF2 significantly reduced IGF2 mRNA levels compared to siCTRL (Fig. S2D of File S1). We previously observed that IGF2 knockdown decreased proliferation in HEY-T30 (Fig. 4B in [7]). Using two other IGF2-targeting siRNA sequences, we confirmed an anti-proliferative effect of IGF2 knockdown, although the effect size varied. In A2780-T15, IGF2 knockdown similarly decreased proliferation. In both Taxol resistant models, IGF2 knock down significantly sensitized cells to Taxol treatment compared to control knock down cells (Fig. 5A). ABCB1 expression was not altered by IGF2 knockdown in HEY-T30 (data not shown).

We next tested whether stable knockdown of IGF2 mRNA could restore sensitivity to Taxol in drug resistant cells and xenografts. The stable IGF2 knockdown cell lines, HEY-T30 shIGF2-p or HEY-T30-shIGF2-v had greater than 70% decrease in IGF2 mRNA compared to HEY-T30 or HEY-T30 shScrambled (Fig. 5B). With regard to protein expression and processing of IGF2, the 21 kDa pre-pro-protein is cleaved into a 15 kDa pro-protein that undergoes several modifications to produce the mature 7 kDa peptide [28]. By Western blot, we confirmed a significant decrease in the 15 kDa pro-IGF2 in HEY-T30 shIGF2-p and shIGF2-v compared to HEY-T30 shScrambled (Fig. S2E of File S1). We did not detect altered levels of the 7 kDa peptide, which is known to be rapidly secreted from the cell [29], and the levels of IGF2 in the conditioned media were below the sensitivity of the ELISA. Unlike the decrease in proliferation after transient knockdown with siRNA, proliferation rates of the stable knockdown cell lines were not significantly altered. The doubling times for HEY-T30 shIGF2-p and shIGF2-v were slightly (non-significantly) longer compared to their cell line of origin, HEY-T30 (Fig. S4 of File S1). This is likely due to the stable selection process favoring expansion of clonal populations able to overcome the anti proliferative effect of IGF2 knockdown through compensatory pathways. The reason that HEY-T30 themselves have a longer doubling time than HEY cells is unclear but appears to be independent of IGF2 expression.

Cytotoxicity assays showed that HEY-T30 shIGF2-p and shIGF2-v were significantly more sensitive to Taxol than HEY-T30 shScrambled or untransfected HEY-T30 (Fig. 5C); HEY-T30 shIGF2-p and sh-IGF2-v were approximately 10-fold and 6-fold more sensitive, respectively, to Taxol than HEY-T30 shScrambled; p<0.01 for both comparisons. In addition, both IGF2 knockdown cell lines were significantly sensitized to the microtubule-interacting drugs ixabepilone and vinblastine (Fig. 5C). There was slight sensitization to doxorubicin (1.6 fold sensitization for either shIGF2-p or sh-IGF2-v compared to shScrambled; p<0.05 for both comparisons, but no change in sensitivity to cisplatin. Given the profound effect of IGF2 knockdown to sensitize cells in tissue culture, we evaluated the effect of IGF2 knockdown *in vivo* in xenograft experiments. Concordant with tissue culture doubling times, the *in vivo* tumor growth prior to treatment initiation was similar between HEY-T30 shIGF2-p and HEY-T30 shScrambled (Fig. S5 of File S1). However, their response to Taxol differed significantly. The growth of IGF2 knockdown xenografts (HEY-T30 shIGF2-p) was significantly suppressed by Taxol similar to the effect observed in sensitive HEY xenografts, while control knockdown xenografts (HEY-T30 shScrambled) remained resistant to Taxol similar to the untransfected HEY-T30 xenografts (Fig. 5D). Two independent experiments were done with concordant findings and the combined data is shown. Thus, IGF2 knockdown restored the efficacy of Taxol in this *in vivo* model of drug resistant human ovarian cancer, consistent with the effect observed in tissue culture. Although the final mean tumor volume in the vehicle control groups differed slightly (shIGF2-p D5W being larger than shScrambled D5W), this difference was not statistically significant at any time points when analyzing either of the 2 independent experiments separately or their combined data. Immunohistochemical analysis of IGF2 protein expression was done in HEY-T30 shIGF2-p xenografts compared to HEY-T30 shScrambled xenografts to confirm that stable knockdown is maintained *in vivo* (Fig. 5E–F).

**Figure 6:**
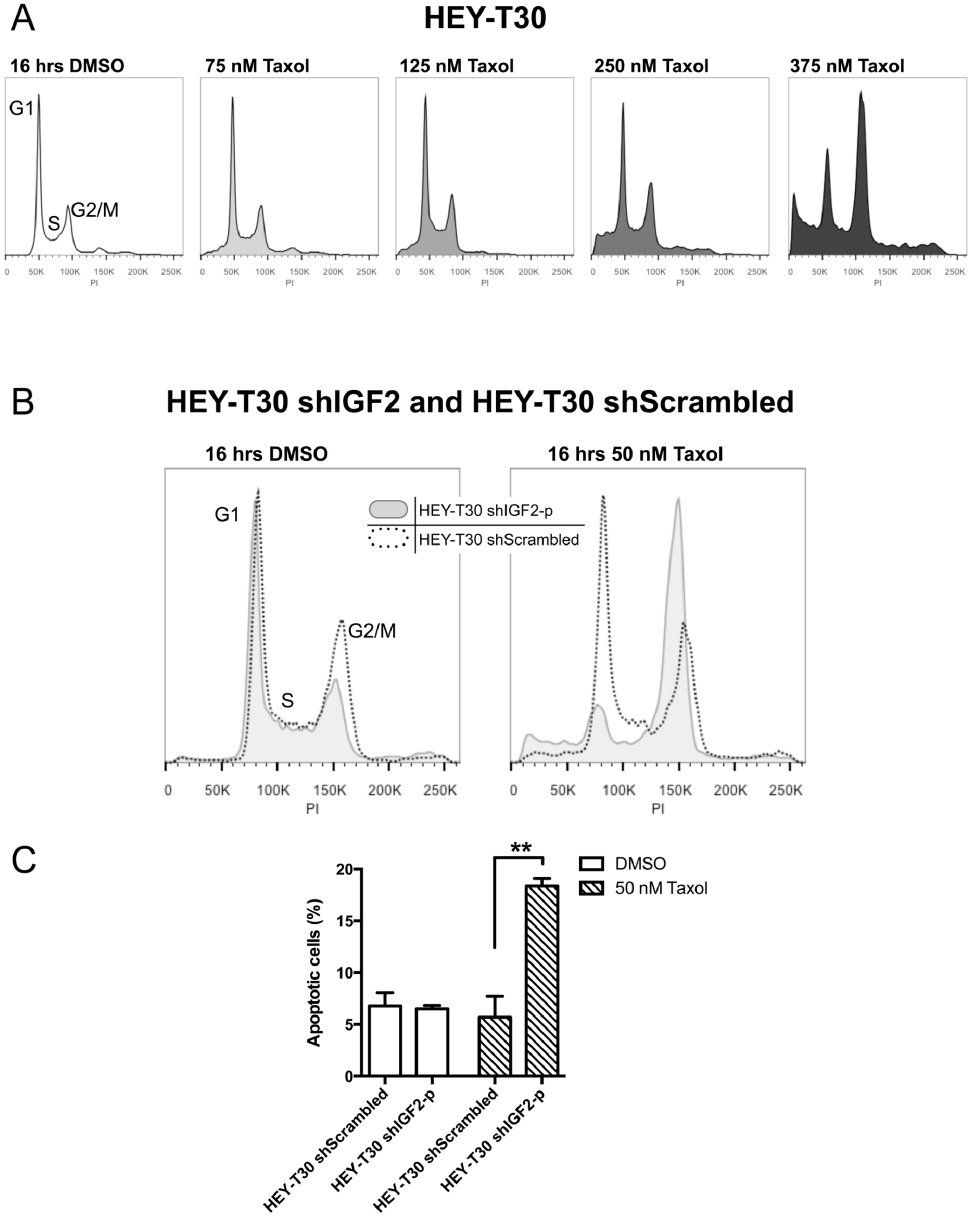
IGF2 knockdown alters the cellular response to Taxol. Taxol-induced G2/M arrest. (A) HEY-T30 cells were treated for 16 hours with the indicated concentrations of Taxol, and DNA content analyzed by flow cytometry. In HEY-T30 cells, G2/M arrest occurred after Taxol treatment at 375 nM but not lower concentrations. (B) shows HEY-T30 shIGF2-p (gray shaded) and HEY-T30 shScrambled (black dotted line) treated with DMSO or with Taxol (50 nM) for 16 hours. The HEY-T30 shIGF2-p cells showed G2/M arrest in response to Taxol while HEY-T30 shScrambled cells did not, at this Taxol concentration. Data from one representative experiment, of two independent experiments, are shown. (C) Apoptosis analysis. Similar to (B), cells were treated with Taxol (50 nM) for 16 hours, harvested and stained with F2N12S (membrane asymmetry marker) and Sytox (dead cell marker), and analyzed by flow cytometry. Cell populations were gated in quadrants for quantitation, where the early apoptotic cell population is delineated by their decreased 585 nm/530 nm ratio due to loss of membrane asymmetry compared to non-apoptotic cells, and are Sytox-negative. Taxol treatment of HEY-T30 shIGF2-p cells resulted in a higher percentage of apoptotic cells compared to treatment with vehicle (DMSO), and Taxol-induced apoptosis was significantly enhanced in HEY-T30 shIGF2 cells compared to HEY-T30 shScrambled, at this time point. **p<0.01; One-way ANOVA with Bonferroni posttest. The bars show the mean±SEM of two independent experiments.

Binding of Taxol to its target on β-tubulin can lead to aberrant mitosis, G2/M arrest, and apoptotic cell death in a dose-dependent, cell-specific manner. To explore the mechanism by which IGF2 knockdown sensitizes cells to Taxol, we examined the cell fates following Taxol treatment of HEY-T30 shIGF2-p compared with HEY-T30 shScrambled. In Taxol sensitive cells, G2/M arrest peaks after 16 to 18 hours of Taxol treatment at concentrations >12 nM [30]. At this time point, Taxol concentrations up to 250 nM did not induce G2/M arrest in HEY-T30. Very high concentrations at or above 375 nM Taxol were required to induce G2/M arrest (Fig. 6A). In contrast, HEY-T30 shIGF2-p, but not HEY-T30 shScrambled, arrested in G2/M when treated with much lower concentrations of Taxol (50 nM) (Fig. 6B). At this same time point, apoptosis analysis using a plasma membrane asymmetry probe (F2N12S, [31]) showed an increase in the early apoptotic population following Taxol treatment in HEY-T30 shIGF2-p but not HEY-T30 shScrambled (Fig. 6C). These data indicate that IGF2 knockdown sensitizes HEY-T30 to Taxol by significantly lowering the drug concentration required to block progression through mitosis and to induce apoptosis.

## Discussion

In this study, we have shown for the first time *in vivo* that IGF2 depletion may be an effective strategy to overcome drug resistance to Taxol. In ovarian cancer patients, high IGF2 expression in tumor tissue is associated with clinically evident drug resistance, as we have now shown in two independent cohorts. Based on the laboratory data described in this paper and previously published data, we propose that IGF2 is a risk factor for disease recurrence and death due to its relationship with resistance to microtubule targeting agents such as Taxol, a drug which is used in first line and recurrence treatment for ovarian cancer. In this manuscript, we have now shown that IGF2 knockdown restores Taxol sensitivity in well-characterized laboratory models of acquired drug resistance.

To reflect the diverse genetic alterations in drug resistant ovarian carcinoma cells [20], [21], six distinct resistant cell lines were profiled and compared to their respective chemosensitive cell lines. All of the drug resistant cell lines had significantly increased IGF2 expression compared to the chemosensitive cell line. Since Taxol is by far the most widely used microtubule-stabilizing agent in clinical use, we focused on the Taxol resistant cell lines for further characterization. Other known mechanisms of Taxol resistance were present in these cell lines, including amplification of *ABCB1* (p-glycoprotein) in the HEY-T30 cells and a *de novo* β-tubulin mutation in the A2780-T15 cells. Despite the multiple genomic alterations in each of these cell lines, our data shows that IGF2 appears to be a significant modulator of their response to Taxol.

Evaluation of different therapeutic strategies revealed that selective inhibition of IGF1R, a major receptor for IGF2, was not sufficient to restore sensitivity to Taxol. Our previous data showed that the small molecule tyrosine kinase inhibitor NVP-AEW541 sensitized drug resistant cells to Taxol; however, we found that in our cell line models, this molecule is not selective for IGF1R, but also inhibits IR. While we specifically excluded p-glycoprotein modulation by NVP-AEW541, we did not perform additional experiments to screen for off-target effects, as this data was previously published [16]. Combining IR knockdown with selective IGF1R blockade did not recapitulate the effect of NVP-AEW541. The lack of efficacy of combined IR knockdown/IGF1R blockade has not been fully elucidated but could be due to residual IR signaling activity (e.g. due to incomplete IR depletion), or IGF2 signaling through alternate, as yet to be determined receptors. Due to the ability of IGF1R, IR, and hybrid receptors to mediate IGF2 signaling [32], [33], we hypothesized that directly targeting the ligand, IGF2, might be a more viable option in clinical development. Therefore, we evaluated the effect of IGF2 depletion via transient or stable knockdown. The proliferation rate in tissue culture and the tumor growth kinetics *in vivo* were similar for stable IGF2 knockdown or control knockdown. Based on tissue culture experiments wherein transient IGF2 knockdown significantly decreased proliferation and cell viability, one might expect that IGF2 knockdown alone would decrease tumor growth of these cells *in vivo*. However, the selection process for stable shIGF2 clones in tissue culture is inherently biased toward selection of transfected cells that retain clonogenic potential. That is, only cells that have developed compensatory mechanisms to overcome the antiproliferative effect of IGF2 knockdown would have been selected for further expansion and subsequently used for the *in vivo* experiments. The significant increase in sensitivity to Taxol in tissue culture was seen after either transient or stable IGF2 knockdown, and translated to restoration of therapeutic efficacy of Taxol treatment of drug resistant xenografts following IGF2 knockdown.

These data provide a critical foundation for developing rational therapeutic strategies to overcome drug resistance by targeting IGF2. In ongoing laboratory studies, we intend to address some additional knowledge gaps. First, Taxol is typically used in combination with platinum agents in the first-line setting, and with both platinum and non-platinum agents in the recurrent setting. In the current study, we focused specifically on Taxol resistance and used well-defined laboratory models of Taxol resistance to avoid confounding the results with platinum-resistance mechanisms or platinum-induced genomic alterations. This proof-of-principle study, demonstrating that IGF2 modulation overcomes multiple Taxol resistance mechanisms, now forms the basis for preclinical testing of IGF2 targeting agents in combination with standard combination ovarian cancer treatment regimens. One example of such an agent is BC-821, a therapeutic vector that expresses diphtheria toxin A under the regulation of an IGF2 fetal promoter [34]. In our subsequent translational studies, we plan to evaluate this IGF2 targeting agent, and others, in combination with Taxol/platinum using prospectively collected primary and recurrent patient-derived xenografts including orthotopic models.

In summary, IGF2 knockdown, but not selective IGF1R inhibition, effectively reverses drug resistance in a Taxol-resistant human ovarian carcinoma xenograft model. The significant association of IGF2 upregulation with drug resistance is observed in genetically diverse cell lines, and its knockdown restores sensitivity to both microtubule-stabilizing and destabilizing agents. Therefore, we suggest that our findings could be applicable to the general problem of resistance to microtubule-targeting chemotherapeutic agents. The clinical relevance of our findings is supported by the validation of IGF2 as a poor prognostic factor for early recurrence and death in ovarian cancer patients. Thus, we have identified IGF2 to be a promising therapeutic target for overcoming drug resistance in ovarian cancer, and further translational studies are merited to bring this fundamental discovery to the clinic.

## Supporting Information

File S1 Contains Table S1, Sequences of primers and oligonucleotides. Figure S1, A2780-T15 β-tubulin mutation. (A) Sequencing data from A2780-T15 show a heterozygous mutation in β-tubulin leading to G360D. (B) This amino acid (red globes) is located in the Taxol-binding pocket of β-tubulin (blue). Taxol is depicted in green. This mutation was not found in HEY-T30. Image made with PyMOL. Figure S2, Knockdown by siRNA and shRNA. (A) IR-A and IR-B mRNA expression, and (B) IGF1R mRNA expression, quantified by reverse transcriptase quantitative PCR, 48 hours after transfection of HEY-T30 and A2780-T15 with IR-targeting siRNA (siIR ) or control nontargeting siRNA (siCTRL). The IR siRNA transfection significantly reduced IR-A and IR-B mRNA levels compared to untransfected (Untreated) or control siRNA (siCTRL) without any significant effect on IGF1R mRNA. Bars show the mean±SEM of at least 3 independent experiments, each done in triplicate. (C) Effect of IR siRNA transfection on Taxol sensitivity. HEY-T30 and A2780-T15 cells were transfected with control nontargeting siRNA (siCTRL) or siRNA targeting IR (siIR), then treated 24 hours later with diluent only (DMSO; solid bars) or Taxol (100 nM for HEY-T30; 22.5 nM for A2780-T15; hatched bars). Seventy-two hours later cells were counted, and surviving fraction calculated as the % cell number relative to untransfected cells treated with diluent only (Untreated; left bar); bars show the mean±SEM of four independent experiments, each done in duplicate. The surviving fraction was significantly reduced following IR siRNA transfection compared to control siRNA in both cell lines. Shown in the hatched bars, the effect of Taxol treatment on HEY-T30 but not A2780-T15 was enhanced in cells transfected with IR siRNA compared with cells transfected with control siRNA. IMC-A12 did not affect the surviving fraction or the response to Taxol in either HEY-T30 or A2780-T15, whether the cells were untransfected, IGF2 siRNA or control siRNA transfected. *p<0.05, **p<0.01, ***p<0.001, ****p<0.0001; One-way ANOVA with Bonferroni posttest. (D) IGF2 mRNA expression, quantified by reverse transcriptase quantitative PCR, 48 hours after transfection of HEY-T30 and A2780-T15 cells with IGF2-targeting siRNA oligonucleotides (siIGF2(1), siIGF2(2), siIGF2(3)) or control nontargeting siRNA (siCTRL). All IGF2 siRNA transfections resulted in at least 80% reduction in IGF2 mRNA compared to the untransfected (Untreated) or control siRNA (siCTRL) transfected cell lines. Bars show the mean±SEM of at least 3 independent experiments, each done in triplicate. (E) IGF2 Western blot. The HEY-T30 shIGF2-p and shIGF2-v cell lines showed a significant decrease in the 15 kDa pro-IGF2 protein compared to HEY-T30, but not in the mature 7 kDa peptide, as determined by densitometry using ImageJ, with normalization to Ponceau staining of protein. A representative blot is shown from four independent experiments, bars show the mean±SEM. Figure S3, Correlation between IGF2 and ABCB1 mRNA. A correlation analysis and graphical representation of reverse transcriptase qPCR data of IGF2 and ABCB1 mRNA. IGF2 mRNA scores of cell lines described in Fig. 1C were used. For ABCB1 mRNA scores were used of cell lines described in Fig. 1D while HEY-B20 (n = 8), HEY-Epo8 (n = 2), A2780-B20 (n = 2), OVCAR-8 (n = 5) and OVCAR-8-D30 (n = 5) were determined similarly by qPCR, where n = number of independent experiments each performed with 3 technical replicates per experiment. The mRNA scores were plotted and correlation calculated. The correlation was not significant (r = 0.6167; p = 0.0857; Spearman correlation). Figure S4, Cell doubling time. Cells were grown in subconfluent monolayers using 6-well dishes, and duplicate wells trypsinized for counting every 24 hours for 96 hours using a Millipore Scepter. Cell doubling time was calculated; bars show the mean±SEM doubling time in hours of at least two independent experiments. The shIGF2-p and shIGF2-v cell lines had a non-significantly longer doubling time than HEY-T30 cells, which in itself had a significant longer doubling time than HEY cells (One-way ANOVA, **p<0.01). Figure S5, Xenograft growth curve of animals from D5W group until first treatment. Female athymic nude mice were subcutaneously injected with 1 million HEY-T30 shScrambled or HEY-T30 shIGF2-p cells and tumors were allowed to grow to an average volume of 120 mm^3^. Data points show the mean tumor volume±SEM for each group at each time point. Two independent experiments were done for a total of 8–10 animals per group. HEY-T30 shIGF2-p xenografts (circle, solid black line); HEY-T30 shScrambled (x, solid purple line). No significant difference in tumor size was observed between shIGF2-p and shScrambled xenografts before treatment started (See Fig. 5D).
(PDF)

File S2 Contains supplementary methods.
(DOC)
