# Supplementary material for: Insulin-Like Growth Factor 2 Silencing Restores Taxol Sensitivity in Drug Resistant Ovarian Cancer
Source: PLoS One. 2014 Jun 16;9(6):e100165. doi: 10.1371/journal.pone.0100165 (PMC4059749; doi:10.1371/journal.pone.0100165)
Supplement: File S1 — Contains Table S1, Sequences of primers and oligonucleotides. Figure S1, A2780-T15 β-tubulin mutation. (A) Sequencing data from A2780-T15 show a heterozygous mutation in β-tubulin leading to G360D. (B) This amino acid (red globes) is located in the Taxol-binding pocket of β-tubulin (blue). Taxol is depicted in green. This mutation was not found in HEY-T30. Image made with PyMOL. Figure S2, Knockdown by siRNA and shRNA. (A) IR-A and IR-B mRNA expression, and (B) IGF1R mRNA expression, quantified by reverse transcriptase quantitative PCR, 48 hours after transfection of HEY-T30 and A2780-T15 with IR-targeting siRNA (siIR ) or control nontargeting siRNA (siCTRL). The IR siRNA transfection significantly reduced IR-A and IR-B mRNA levels compared to untransfected (Untreated) or control siRNA (siCTRL) without any significant effect on IGF1R mRNA. Bars show the mean±SEM of at least 3 independent experiments, each done in triplicate. (C) Effect of IR siRNA transfection on Taxol sensitivity. HEY-T30 and A2780-T15 cells were transfected with control nontargeting siRNA (siCTRL) or siRNA targeting IR (siIR), then treated 24 hours later with diluent only (DMSO; solid bars) or Taxol (100 nM for HEY-T30; 22.5 nM for A2780-T15; hatched bars). Seventy-two hours later cells were counted, and surviving fraction calculated as the % cell number relative to untransfected cells treated with diluent only (Untreated; left bar); bars show the mean±SEM of four independent experiments, each done in duplicate. The surviving fraction was significantly reduced following IR siRNA transfection compared to control siRNA in both cell lines. Shown in the hatched bars, the effect of Taxol treatment on HEY-T30 but not A2780-T15 was enhanced in cells transfected with IR siRNA compared with cells transfected with control siRNA. IMC-A12 did not affect the surviving fraction or the response to Taxol in either HEY-T30 or A2780-T15, whether the cells were untransfected, IGF2 siRNA or control siRNA transfected. [file pone.0100165.s001.pdf]

Table S1

Target	Forward	Reverse	Efficiency	Amplicon Size (bp)	Accession Number
IGF2 mRNA	ACCGTGCTTCCGGACAACT	TGGACTGCTTCCAGGTGTCA	102.5%	73	NM_000612.4
IGF2 gDNA	GGTGCTAACACGGCTCTCTC	CGGAAACAGCACTCCTCAAC	92.1%	100	NM_000612.4
ABCB1 mRNA	GTCAGCTGCTGTCTGGGCAAAGATAC	TGCTGCCAAGACCTCTTCAGCTACT	102.0%	100	NM_000927.3
ABCB1 gDNA	TTGAAGGAAAAGCAAATCTTCC	TTGTCAAGCCAATTTGAATAGC	103.6%	98	NM_000927.3
PPIB mRNA	AAGTCACCGTCAAGGTGTATTTT	GATCACCCGGCCTACATCTTC	91.4%	62	NM_000942.4
ALB gDNA	TCCTGACCAAGCTTAACCAGTAT	CCAAAAAGGGTATGCTAAATGG	98.8%	117	NM_000477
IGF1R	TGAAAGTGACGTCCTGCATTTC	GGTACCGGTGCCAGGTTATG	94.0%	92	NM_000875.3
IR-B	TGAAGGAGCTGGAGGAGTCCTCG	CCTAGGGTCCTCGGCACCAGTG	115.0%	110	NM_000208.2
IR-A	CGTCCCCAGGCCATCTCGG	GCTGGTCGAGGAAGTGTTGGGG	117.5%	103	NM_001079817.1

Target	Forward	Reverse
IGF2 siRNA #1	AAGGUGAGAAGCACCAGCAUCGACU	AAGGUGAGAAGCACCAGCAUCGACU
IGF2 siRNA #2	UCGCCUCGUGCUGCAUUGCUGCUUA	UAAGCAGCAAUGCAGCACGAGGCGA
IGF2 siRNA #3	CGUGGCAUCGUUGAGGAGUGCUGUU	AACAGCACUCCUCAACGAUGCCACG
IR siRNA	CUAGUCCUGCAGAGGAUUU	AAAUCCUCUGCAGGACUAG
IGF2 shRNA	CACCGCGTGGCATCGTTGAGGAGTGCTGTTCGAAAACAGCACTCCTCAACGATGCCACG	AAAACGTGGCATCGTTGAGGAGTGCTGTTTTCGAACAGCACTCCTCAACGATGCCACGC
Scrambled shRNA	CACCGGGTGCTCGTGTCGCATGTAGTGTACGAATACACTACATGCGACACGAGCACCC	AAAAGGGTGCTCGTGTCGCATGTAGTGTATTCGTACACTACATGCGACACGAGCACCC

Figure S1

A

A2780-T15 ß-tubulin mutation

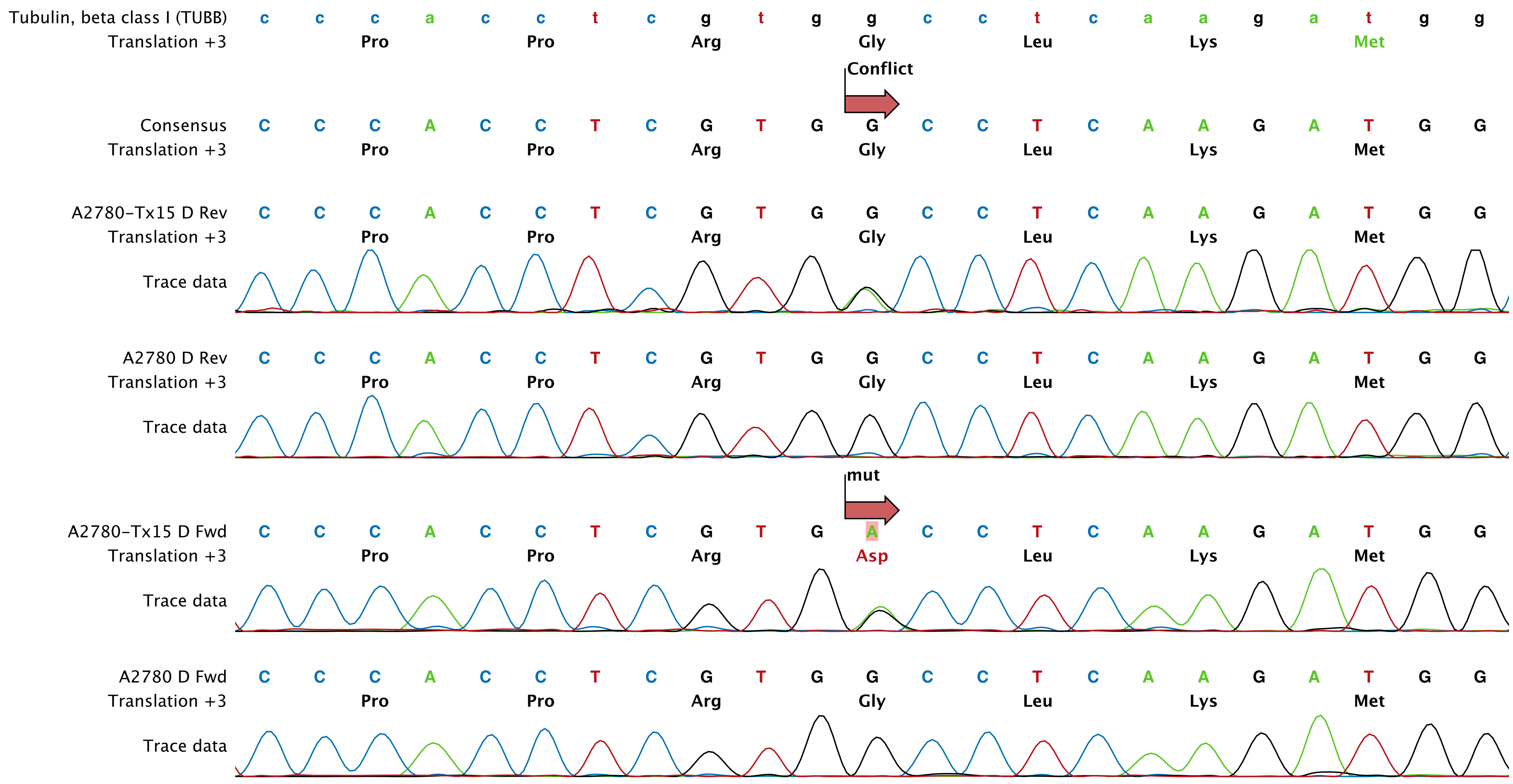

B

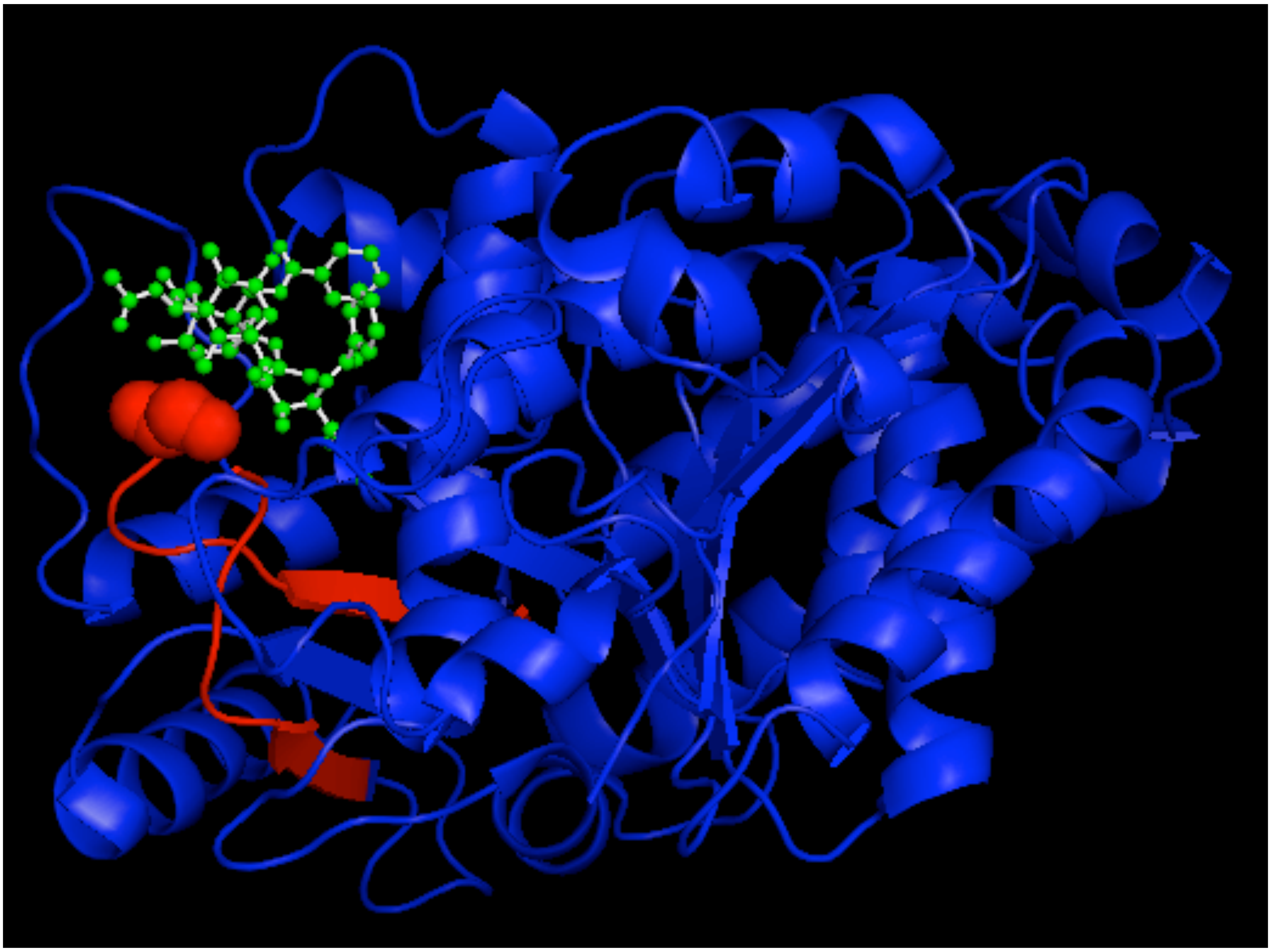

Figure S2

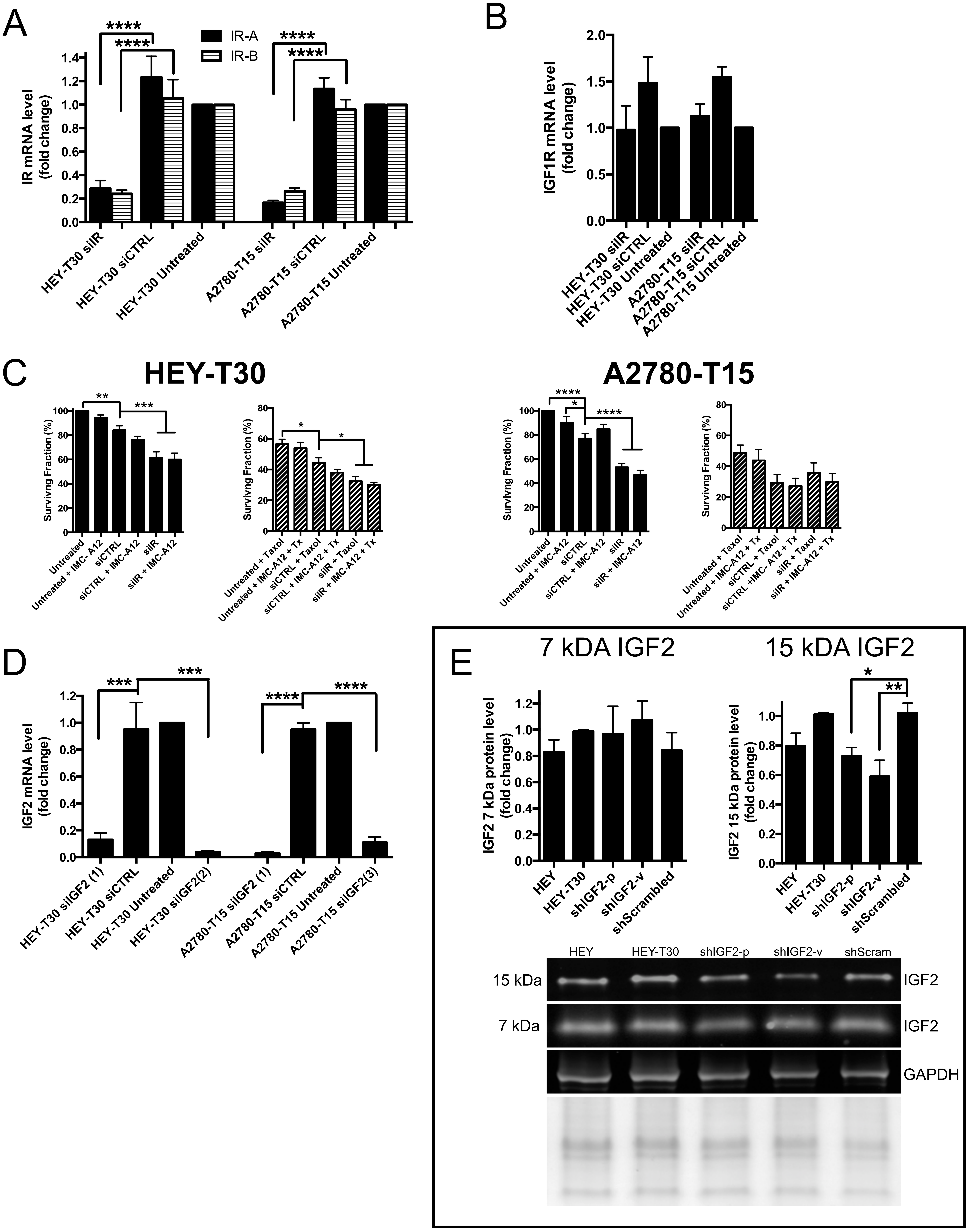
A
IR-A
IR-B
IR mRNA level (fold change)
HEY-T30 siIR
HEY-T30 siCTRL
HEY-T30 Untreated
A2780-T15 siIR
A2780-T15 siCTRL
A2780-T15 Untreated
B
IGF1R mRNA level (fold change)
C
HEY-T30
A2780-T15
Surviving Fraction (%)
Untreated
Untreated + IMC-A12
siCTRL
siCTRL + IMC-A12
siIR
siIR + IMC-A12
Untreated + Taxol
Untreated + IMC-A12 + Tx
siCTRL + Taxol
siCTRL + IMC-A12 + Tx
siIR + Taxol
siIR + IMC-A12 + Tx
D
IGF2 mRNA level (fold change)
HEY-T30 siIGF2 (1)
HEY-T30 siCTRL
HEY-T30 Untreated
HEY-T30 siIGF2(2)
A2780-T15 siIGF2 (1)
A2780-T15 siCTRL
A2780-T15 Untreated
A2780-T15 siIGF2(3)
E
7 kDA IGF2
15 kDA IGF2
IGF2 7 kDa protein level (fold change)
IGF2 15 kDa protein level (fold change)
HEY
HEY-T30
shIGF2-p
shIGF2-v
shScrambled
shScram
15 kDa
7 kDa
IGF2
GAPDH

Figure S3

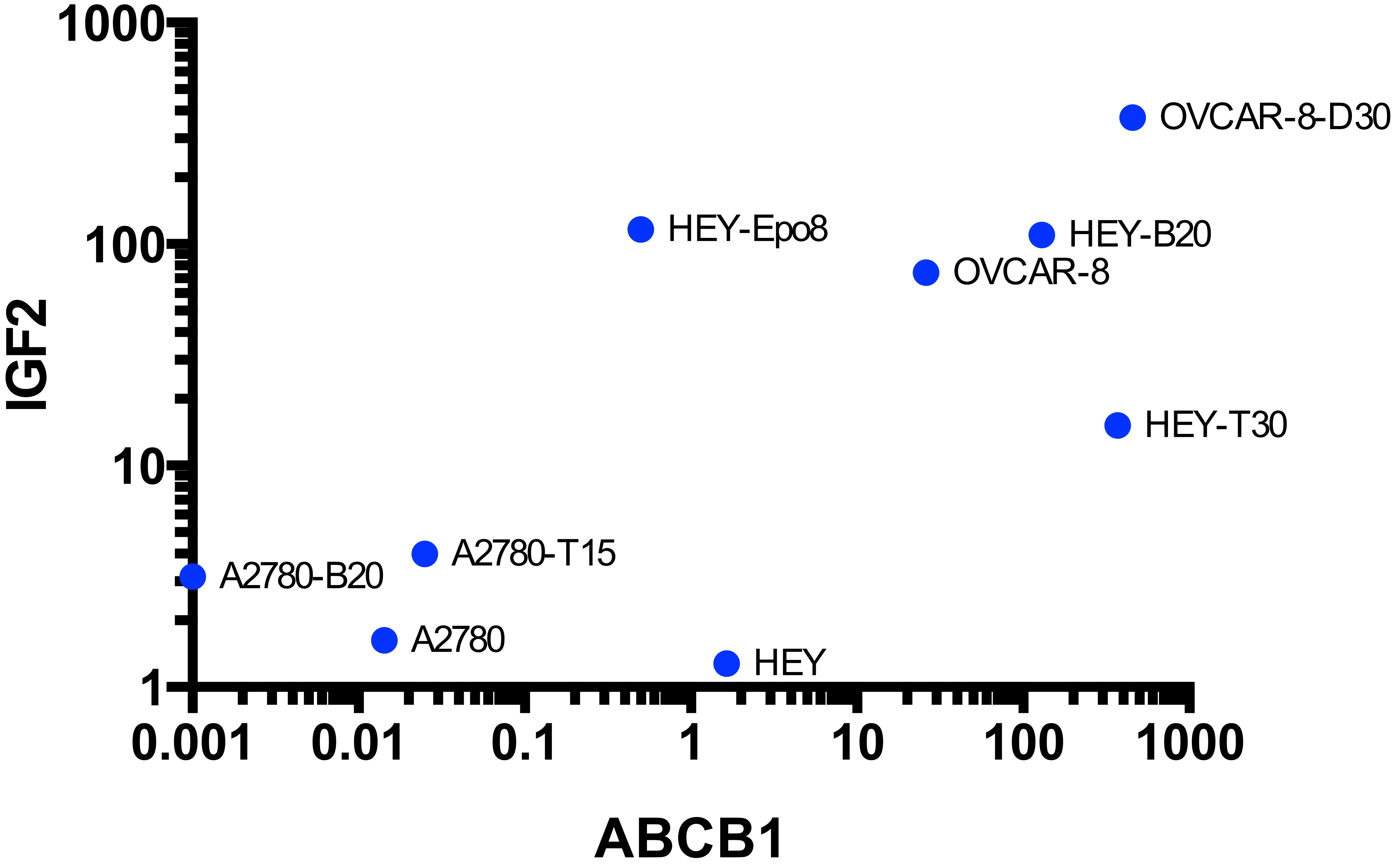

IGF2
1000
100
10
1
0.001
0.01
0.1
1
10
100
1000
ABCB1
OVCAR-8-D30
HEY-Epo8
HEY-B20
OVCAR-8
HEY-T30
A2780-T15
A2780-B20
A2780
HEY

Figure S4

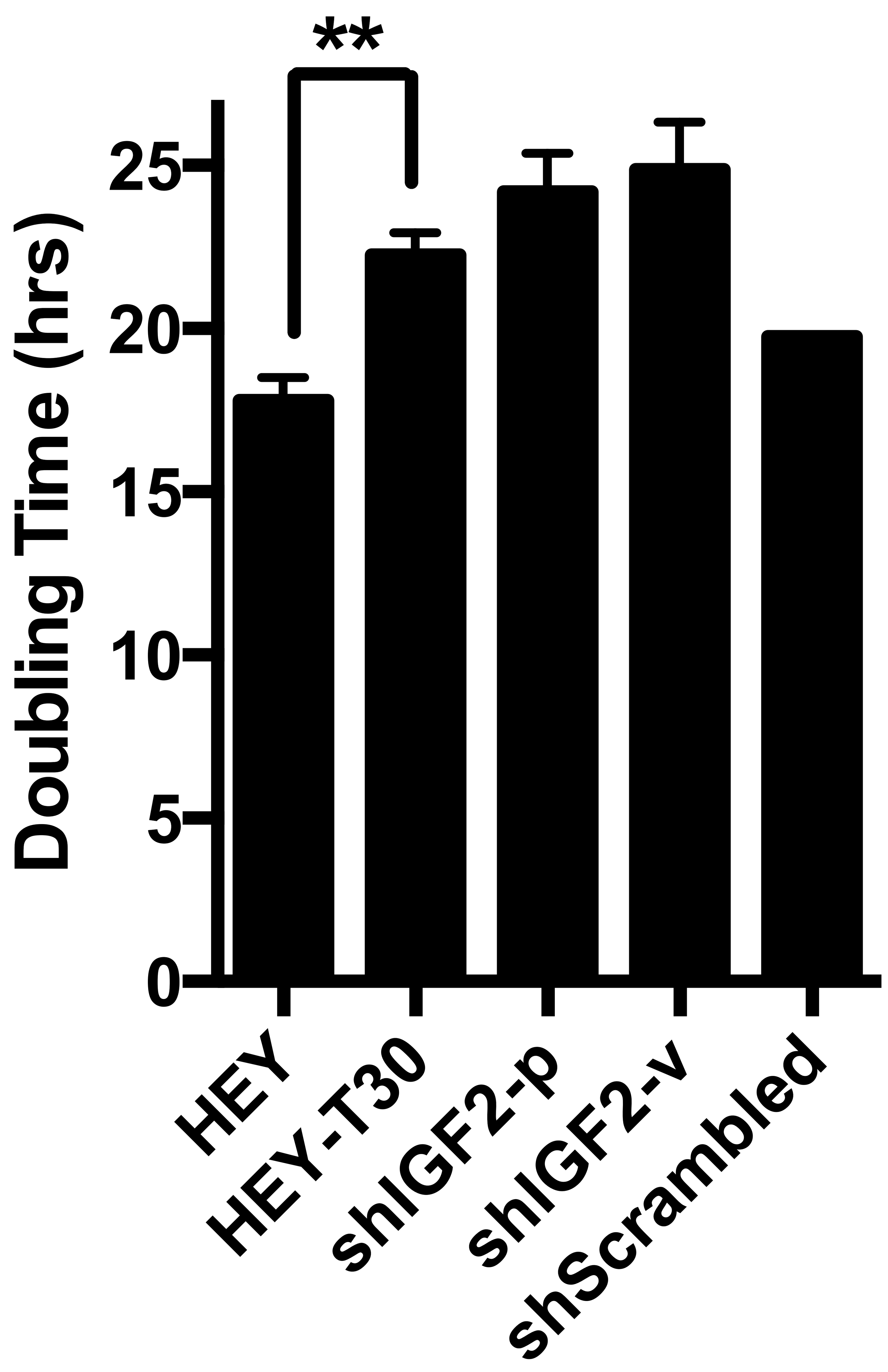

**
Doubling Time (hrs)
0
5
10
15
20
25
HEY
HEY-T30
shIGF2-p
shIGF2-v
shScrambled

Figure S5

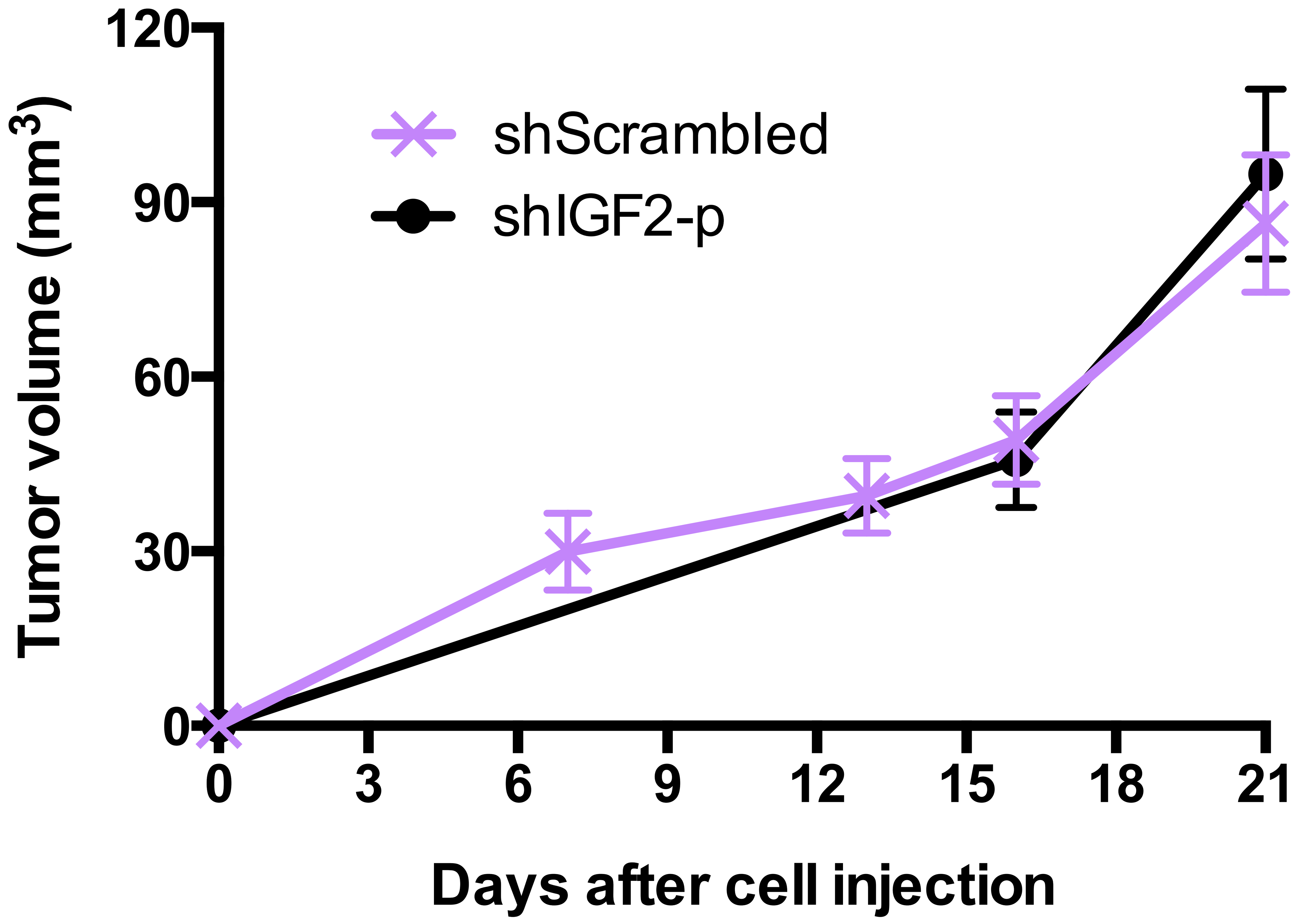

shScrambled
shIGF2-p
Tumor volume (mm3)
120
90
60
30
0
0
3
6
9
12
15
18
21
Days after cell injection
