# Supplementary material for: Insulin-Like Growth Factor 2 Silencing Restores Taxol Sensitivity in Drug Resistant Ovarian Cancer
Source: PLoS One. 2014 Jun 16;9(6):e100165. doi: 10.1371/journal.pone.0100165 (PMC4059749; doi:10.1371/journal.pone.0100165)
Supplement: File S2 — Contains supplementary methods. (DOC) [file pone.0100165.s002.doc]

Supplementary Methods:

*Sequencing of ß-tubulin*

Complementary DNA of A2780 and A2780-T15 was made as described for reverse transcriptase qPCR. Using four primer sets spanning the whole ß-tubulin mRNA, a PCR with 250 ng cDNA was run. The products were run on an agarose gel, the bands cut and the DNA extracted with a QIAquick Gel Extraction Kit (Qiagen). The DNA was submitted to the in-house DNA sequencing facility and the resulting sequences analyzed using Genomic Workbench with NM_178014 (TUBB) as the template. PyMOL was used to model the found mutation.

*Western blot of IGF2*

Western blot for IGF2 was performed similarly to Western blots described in the main methods, except using tris-tricine gels and anti-human IGF2 antibody 1:5000 (Abcam ab9574) or GAPDH (Cell Signal).
